# Anti-inflammatory dietary patterns and cognitive aging in older adults: a scoping review of mechanistic, clinical, and nursing implications

**DOI:** 10.3389/fnut.2026.1952047

**Published:** 2026-09-18

**Authors:** Chunli Dong, Jingya Tai, Yitian Ma, Yuchen Wang, Junxiao Yang

**Affiliations:** 1 School of Nursing, Shandong First Medical University & Shandong Academy of Medical Sciences, Jinan, China; 2 School of Nursing, Shenyang Medical College, Shenyang, China; 3 School of Nursing, Jining Medical University, Jining, China

**Keywords:** anti-inflammatory diet, cognitive aging, dementia prevention, dietary inflammatory index, neuroinflammation, scoping review

## Abstract

**Background:**

Dietary patterns with lower inflammatory potential may support cognitive aging, but Mediterranean, Dietary Approaches to Stop Hypertension (DASH), Mediterranean–DASH Intervention for Neurodegenerative Delay (MIND), and dietary inflammatory scores represent non-equivalent exposures, and observational findings have not been consistently confirmed in randomized trials. This scoping review mapped evidence linking whole-diet patterns and dietary inflammatory indices with cognitive aging, measured biological pathways, and clinical interpretation and potential nursing implications.

**Methods:**

PubMed, Embase, Web of Science Core Collection, and Scopus were searched from inception to 30 June 2026. Eligible studies evaluated dietary patterns or validated inflammatory indices in older adults or cohorts assessed in later life and reported cognitive, dementia, mechanistic, neuroimaging, neuropathological, or implementation outcomes. Evidence was synthesized narratively, with observational and interventional findings considered separately.

**Results:**

Thirty-eight reports were included: 27 longitudinal observational studies, seven randomized intervention reports, three cross-sectional biomarker or neuroimaging studies, and one *post hoc* analysis. Mediterranean- and MIND-oriented patterns and lower dietary inflammatory potential were recurrently associated with more favorable cognitive outcomes, but findings varied across definitions, populations, measures, and follow-up. Randomized evidence was mixed and not definitive. Fourteen reports measured inflammatory, proteomic, microbiome, neuroimaging, cerebrospinal-fluid, spectroscopy, or neuropathological outcomes, supporting selected biological links without establishing a complete diet-neuroinflammation-cognition pathway. No study evaluated a complete nurse-led or nurse-supported dietary pathway; the nursing framework is author-derived and untested.

**Conclusion:**

The evidence is epidemiologically suggestive and biologically plausible, but does not establish that any specific diet prevents dementia. Dietary improvement may be incorporated into individualized multidomain risk reduction, provided that nutritional adequacy, frailty, cultural fit, affordability, and sustained adherence are addressed. Better-contrasted trials, temporally ordered mechanistic studies, and prospective implementation evaluations are needed.

## Introduction

Population aging is making cognitive decline and dementia a major clinical and public-health challenge. The Global Burden of Disease Study estimated that 57.4 million people were living with dementia in 2019 and projected this number to reach 152.8 million by 2050 (1). Progressive impairment in memory, executive function, and judgment can compromise medication management, nutrition, mobility, and other aspects of self-care, while increasing caregiver dependence and health-system demand. The 2024 Lancet Commission estimated a combined population-attributable fraction of 45.3% for 14 potentially modifiable risk factors (2). Although diet was not modeled as a separate component of that fraction, it intersects with several established risk domains, including hypertension, dyslipidemia, obesity, diabetes, and vascular health. Diet may also influence chronic low-grade inflammation, or “inflammaging,” which can link peripheral immune and metabolic disturbances with blood–brain barrier dysfunction, neurovascular injury, maladaptive microglial responses, synaptic damage, and amyloid- and tau-related pathology (3, 4). Dietary modification therefore sits at an important intersection between population-level risk reduction and the biology of brain aging.

Research has consequently shifted from isolated nutrients toward whole-diet patterns and multidimensional measures of dietary inflammatory potential. Mediterranean, Dietary Approaches to Stop Hypertension (DASH), Mediterranean–DASH Intervention for Neurodegenerative Delay (MIND), and other plant-forward patterns assess adherence to predefined combinations of foods. By contrast, the Dietary Inflammatory Index (DII) and energy-adjusted Dietary Inflammatory Index (E-DII) apply literature-derived inflammatory weights, whereas the empirical dietary inflammatory index (EDII) and empirical dietary inflammatory pattern (EDIP) derive food-based weights from associations with circulating inflammatory biomarkers (5, 6). These constructs overlap, but they are not interchangeable. A dietary pattern associated with healthier cognition may operate through vascular, metabolic, antioxidant, or behavioral pathways that are only partly represented by an inflammatory score. The evidence base has also expanded beyond single dietary assessments and brief cognitive screens to include repeated exposure measurement, long-term cohorts, randomized trials, structural neuroimaging, cerebrospinal fluid (CSF) biomarkers, proteomics, inflammatory panels, microbiome profiling, mediation analysis, and magnetic resonance spectroscopy (7). Recent human studies have linked dietary exposures with dementia risk, brain structure, immune-related plasma proteins, circulating inflammatory mediators, microbial signatures, and neuroinflammation-associated metabolites (8–12). Even so, greater biological resolution has not resolved the question of causality. In a 3-year randomized trial, cognition and magnetic resonance imaging (MRI) outcomes did not differ significantly between the MIND-diet and active-control groups (13), highlighting the continuing importance of exposure overlap, adherence convergence, intervention timing, and disease stage.

Several reviews have synthesized important parts of this literature. Broad systematic reviews have examined dietary patterns and cognitive health, pattern-specific reviews have focused on the MIND diet, and intervention reviews have evaluated whole-diet effects on memory and cognition in healthy older adults (14–16). A broader synthesis has also integrated observational and randomized evidence and outlined priorities for future dietary trials (7). Collectively, these reviews support biological and epidemiological plausibility, while showing that observational associations are generally more consistent than randomized findings and that results depend on dietary definition, population, comparator, cognitive measure, and follow-up duration. However, most existing syntheses remain organized around a single dietary pattern, study design, or outcome category. Mechanisms are often discussed as plausible explanations rather than examined as directly measured human pathways. Literature-derived and empirically derived inflammatory indices may also be considered alongside healthy dietary-pattern scores without sufficient attention to their conceptual differences. Less consideration has been given to how dietary measurement, intervention contrast, and implementation conditions affect clinical interpretation. What remains missing is an integrated account linking the dietary construct, measured biology, cognitive endpoint, and strength of inference.

The central question is therefore not simply whether a “healthy” diet is associated with better cognition, but whether dietary inflammatory potential is linked to cognitive aging through pathways demonstrated in humans, in which populations, and under what conditions. Answering this question requires distinctions between correlation, moderation, mediation, and intervention effects; between directly measured biological changes and mechanistic proxies; between peripheral inflammation and central neuroinflammation; and between diet-specific effects and broader lifestyle or cardiometabolic influences. Human proteomic, cytokine, microbiome, imaging, and spectroscopy studies have identified candidate pathways, but the evidence remains uneven. Few studies have established a temporal sequence from dietary exposure to biological change and subsequent cognitive outcome, while direct assessment of microglial activation and oxidative-stress mediation in older-adult whole-diet studies remains particularly limited (9–12).

Clinical translation introduces a different set of constraints. Dietary recommendations for older adults must be considered alongside malnutrition risk, comorbidity, medication burden, food access, cultural preferences, functional capacity, caregiver support, and the ability to sustain behavioral change (17). These conditions are seldom examined together with mechanistic and cognitive evidence. They are particularly relevant to nursing practice because nurses contribute to nutritional and cognitive screening, practical education, adherence support, caregiver engagement, longitudinal monitoring, and referral coordination across community and clinical settings. However, the evidence needed to combine these functions into a reproducible, prospectively evaluated nurse-supported pathway remains limited.

We therefore conducted a systematic scoping review to map human evidence on defined whole-diet patterns and dietary inflammatory indices in older adults; compare observational and interventional findings across cognitive decline, cognitive impairment, mild cognitive impairment (MCI), dementia, and related brain outcomes; and chart biological pathways that were directly measured in the included studies. We also examined methodological and translational barriers and developed an author-derived, evidence-informed framework for potential nursing roles encompassing screening, risk stratification, individualized planning, adherence support, caregiver involvement, longitudinal monitoring, and interprofessional referral. By separating demonstrated associations from inferred mechanisms and evidence-supported clinical actions from untested implementation models, this review aims to identify which findings may cautiously inform practice and where prospective mechanistic and implementation research remains necessary.

## Methods

### Review design

This study was conducted as a systematic scoping review. A scoping-review design was selected because the literature spanned observational cohorts, randomized and nonrandomized dietary interventions, cross-sectional studies, neuroimaging investigations, biomarker and proteomic analyses, microbiome research, and implementation-related evidence. The purpose was to characterize the breadth, composition, and methodological heterogeneity of the evidence, map the biological pathways that had been directly examined, and identify clinical and translational gaps. The review was therefore not designed primarily to estimate a single pooled intervention effect.

The review questions were structured using the Population-Concept-Context framework. The population comprised older adults or cohorts in which cognitive outcomes were assessed in later life. The central concept was the inflammatory potential of diet, including defined anti-inflammatory or pro-inflammatory dietary patterns, validated dietary inflammatory indices, and their relationships with cognitive aging and directly measured biological pathways. Eligible contexts included community, outpatient, research-cohort, residential-care, and other clinical settings. The review addressed three questions: what evidence links dietary inflammatory potential or defined dietary patterns with cognitive function, cognitive decline, MCI, and dementia; which inflammatory, gut-brain, neurovascular, neuroimaging, molecular, or neuropathological pathways have been directly measured; and what findings are relevant to clinical and nursing assessment, counseling, adherence support, and longitudinal monitoring.

The review was guided by established methodological recommendations for scoping reviews and reported in accordance with the Preferred Reporting Items for Systematic Reviews and Meta-Analyses Extension for Scoping Reviews (PRISMA-ScR) (18–20). Reporting of the literature search was also informed by the Preferred Reporting Items for Systematic Reviews and Meta-Analyses literature search extension (PRISMA-S) (21). The review was not prospectively registered.

### Information sources and search strategy

PubMed, Embase, Web of Science Core Collection, and Scopus were searched from database inception to 30 June 2026. Across databases, the search strategy covered three core concepts: dietary exposure, cognitive or brain-aging outcomes, and older populations. Dietary terms included anti-inflammatory diet, inflammatory diet, Dietary Inflammatory Index, energy-adjusted Dietary Inflammatory Index, empirically derived inflammatory dietary patterns, Mediterranean diet, MIND diet, DASH diet, plant-based or plant-forward diet, dietary pattern, omega-3 fatty acids, polyphenols, flavonoids, and dietary fiber. The component terms were used only to expand retrieval sensitivity because whole-diet records may mention such components in titles, abstracts, or indexing; they did not make nutrient-, supplement-, or bioactive-only studies eligible for the final evidence set. Cognitive terms included cognitive decline, cognitive impairment, mild cognitive impairment, dementia, Alzheimer disease, cognitive aging, brain aging, and cognitive frailty. Population terms included aging, ageing, elderly, older adults, and older people.

Database-specific strategies were adapted to the indexing structure and retrieval characteristics of each database. PubMed used the broader diet-cognition-aging framework. In Embase, Web of Science, and Scopus, an additional mechanism-related block was combined with the core concepts as an AND-based precision filter for the mechanistic component. This was intended to improve specificity, but it could also reduce sensitivity for eligible diet-cognition reports that lacked mechanistic indexing. The mechanism block was not an eligibility criterion at full-text screening; eligibility was determined from the prespecified population, dietary-exposure, and outcome criteria. Because the database strategies were asymmetric, equal sensitivity across databases cannot be assumed, and this limitation was considered when interpreting the completeness of the evidence map. Free-text terms were combined with controlled vocabulary where available, with syntax, field restrictions, phrase searching, truncation, and subject headings adapted to each database.

Search development was informed by the Peer Review of Electronic Search Strategies guideline and PRISMA-S reporting recommendations (21, 22). The complete database-specific strategies and the number of records retrieved are provided in Supplementary Table S1.

### Eligibility criteria

Studies were eligible when they included adults aged 60 years or older, populations explicitly described by the original investigators as older adults, or mixed-age samples from which findings for older participants could be separately extracted. Longitudinal cohorts that began in midlife were also eligible when dietary exposure was assessed prospectively and the mean or median age at cognitive-outcome assessment was at least 60 years, or when the study explicitly evaluated cognition or dementia during later life. No restrictions were placed on sex, country, ethnicity, residential setting, or baseline cognitive status. Baseline cognitive status was nevertheless recorded so that findings from cognitively unimpaired participants, people with subjective cognitive decline or MCI, and people with established dementia were not interpreted as equivalent stages of prevention.

Eligible exposures included explicitly defined anti-inflammatory or pro-inflammatory diets, validated dietary inflammatory indices, and reproducibly defined whole-diet patterns. These included the DII, E-DII, EDII, EDIP, Mediterranean diet, MIND diet, DASH diet, and plant-forward dietary patterns. The dietary exposure had to be a principal analytical variable and had to be described sufficiently to identify the scoring system, intervention content, or food-pattern definition. Studies focused solely on isolated nutrients, supplements, or food-derived bioactive compounds were not eligible for the final evidence set, including as stand-alone mechanistic evidence. Corresponding component terms were retained only to improve search sensitivity, and none of the 38 included reports was an isolated nutrient-, supplement-, or bioactive-only study.

A study was required to report at least one eligible outcome. Clinical outcomes included objectively assessed global or domain-specific cognitive function, cognitive decline, cognitive impairment, MCI, cognitive frailty, incident dementia, or Alzheimer disease. Mechanistic outcomes included circulating inflammatory or immune markers, intestinal-barrier markers, gut-microbiome features, neuroinflammatory metabolites, structural or diffusion neuroimaging, white-matter injury, brain atrophy, amyloid or tau biomarkers, neuropathology, neurovascular changes, or other directly measured indicators of brain aging. Mechanistic studies without a clinical cognitive outcome were eligible only when the measured marker had an explicit and prespecified relevance to cognitive aging or neurodegeneration. Implementation-related outcomes, including recruitment, adherence, feasibility, acceptability, safety, caregiver involvement, intervention fidelity, and dietary-delivery procedures, were eligible when they were reported in relation to a defined dietary intervention or counseling approach in the target population.

Eligible designs included randomized and nonrandomized dietary interventions, prospective and retrospective cohort studies, case–control studies, analytical cross-sectional studies, biomarker and neuroimaging studies, qualitative or mixed-methods studies, and implementation studies. Only original, peer-reviewed, English-language full-text articles with sufficient information on the population, dietary exposure, outcome assessment, and principal findings were included.

Studies were excluded when the dietary exposure was inadequately defined, was not a principal analytical variable, or was limited to enteral or parenteral nutrition, tube-feeding formulations, critical-care nutritional support, or pharmacological anti-inflammatory treatment. Studies addressing acute or non-age-related cognitive disorders, including delirium, postoperative cognitive dysfunction, acute brain injury, infectious encephalopathy, or chemotherapy-related cognitive impairment, were excluded. Reports limited to peripheral metabolic outcomes, such as body weight, glycemia, or lipid concentrations, without an eligible cognitive, neuroinflammatory, neuroimaging, neuropathological, or implementation outcome were also excluded.

Multicomponent lifestyle interventions were excluded from the clinical-effectiveness synthesis when the independent contribution of diet could not be determined. A dietary contribution was considered separately extractable when the study included a distinct diet-only arm, applied the same non-dietary co-interventions across comparison groups, or reported a prespecified analysis specifically relating dietary exposure or adherence to an eligible outcome. Multicomponent studies could still contribute to the implementation synthesis when diet-specific information on delivery, adherence, feasibility, caregiver participation, or nursing practice was available. Animal studies, *in vitro* studies, case reports, small descriptive case series, study protocols, conference abstracts, editorials, commentaries, reviews, duplicate publications, retracted articles, and reports without accessible full text or sufficient data were excluded from the formal evidence set. Animal or laboratory literature, when cited for context, was not counted among the included studies.

### Study selection

We imported all records into EndNote 2025, removed duplicates electronically with manual verification, and used two reviewers to screen titles and abstracts and then full texts independently. Records considered potentially eligible by either reviewer advanced to full-text assessment. Disagreements were resolved by discussion or, when required, consultation with a third reviewer. The eligible report served as the unit of inclusion. Site-specific or secondary reports of randomized trials were eligible when they reported an eligible cognitive outcome among participants allocated within the parent trial; reports from the same parent trial were linked as one study family and were not interpreted as independent confirmation. We recorded one principal reason for each full-text exclusion and summarized the selection process in a PRISMA-ScR flow diagram.

### Data extraction

We developed a standardized extraction form aligned with the review questions and planned evidence tables. We extracted publication and design characteristics, cohort or setting, participant characteristics and baseline cognitive status, sample size and age, dietary pattern or inflammatory index, dietary assessment and exposure definition, comparator or intervention content, follow-up, cognitive outcomes and instruments, biological or neuroimaging measures, implementation outcomes, covariates, and principal findings. Association estimates, randomized intervention effects, mediation estimates, moderation or interaction effects, and directly measured mechanistic findings were coded in separate fields rather than merged into a single evidence category. Effect estimates were charted only as reported, including hazard ratios, odds ratios, risk ratios, regression coefficients, mean differences, 95% confidence intervals, *p* values, and interaction or mediation estimates. We retained the original analytical scale and did not impute or infer missing numerical results. The extraction process followed current recommendations for scoping-review data charting and presentation (19, 23).

One reviewer completed the initial extraction and a second checked every entry against the full text. We prioritized the analysis designated as primary by the original authors and, for randomized trials, between-group intention-to-treat findings. All eligible outcomes were charted, while the main characteristics table emphasized the principal outcome of each report.

### Design-specific methodological appraisal

Because the purpose of the review was to map the evidence rather than exclude studies according to a common quality threshold, no formal tool-based risk-of-bias classification, overall methodological score, or certainty-of-evidence rating was undertaken. Instead, design-specific bias and validity domains were prespecified and used to qualify the narrative interpretation of evidence maturity and consistency.

Prespecified domains included participant selection and representativeness, sample size, duration and completeness of follow-up, validity and timing of dietary measurement, validity and sensitivity of cognitive assessment, temporal ordering, control of confounding, attrition and missing data, multiplicity, analytical appropriateness, reverse causation, selection bias, and generalizability. For randomized studies, additional attention was given to randomization, exposure contrast, contamination, adherence, attrition, and whether conclusions were based on intention-to-treat or post-randomization analyses. For biomarker, neuroimaging, proteomic, and microbiome studies, the assessment considered sample selection, timing of exposure and outcome measurement, multiple testing, external validation, and whether the proposed biological pathway had been directly measured rather than inferred. These domains were considered within linked study families so that multiple reports from one cohort or trial did not create the appearance of independent replication.

These features were used to contextualize the narrative synthesis and identify recurrent methodological limitations; they were not combined into an overall score, used to assign risk-of-bias categories, or applied as exclusion criteria.

### Evidence synthesis

Evidence was synthesized narratively and presented in structured tables and evidence maps. Studies were charted by publication year, country, design, population, dietary exposure, follow-up, and outcome domain. Whole-diet pattern scores (Mediterranean, MIND, DASH, and related patterns) and validated dietary inflammatory indices (DII, E-DII, EDII, and EDIP) were maintained as separate exposure streams and were neither pooled nor treated as equivalent measures of inflammation. Observational studies were interpreted separately from randomized and nonrandomized interventions.

Mechanistic evidence was initially organized according to systemic inflammatory, gut brain, neurovascular, neuroimaging, molecular, and neuropathological domains. These categories were refined iteratively during data charting to reflect the directly measured pathways identified in eligible reports. Pathways that were biologically plausible but not directly measured were treated as evidence gaps rather than empirical findings.

We did not conduct a meta-analysis because dietary definitions, scoring systems, populations, designs, cognitive instruments, biological outcomes, follow-up, exposure contrasts, and statistical measures differed substantially. Consistency was assessed descriptively at the study-family level according to direction of association or effect, uncertainty around estimates when reported, persistence across related analyses, concordance across independent study families and study designs, and whether a proposed pathway was directly measured. Statistical significance alone was not considered sufficient evidence of consistency, and multiple reports from the same cohort or intervention were not counted as independent confirmations. The synthesis emphasized evidence distribution, contrasts between observational and intervention findings, direct mechanistic testing, and methodological and translational gaps.

Implementation-related findings were charted according to recruitment, retention, adherence, feasibility, acceptability, safety, caregiver involvement, intervention delivery, follow-up, and referral-related elements. During synthesis, recurring care functions were grouped iteratively into five domains: screening, risk stratification, individualized planning, adherence support, and longitudinal monitoring and care coordination. These domains were used only to construct an author-derived organizational framework for potential nurse-supported roles within multidisciplinary care. The framework was not treated as an intervention, clinical pathway, guideline, or standard of care, and elements without direct empirical evaluation were explicitly identified as proposed.

## Results

### Study selection

**Figure 1:**
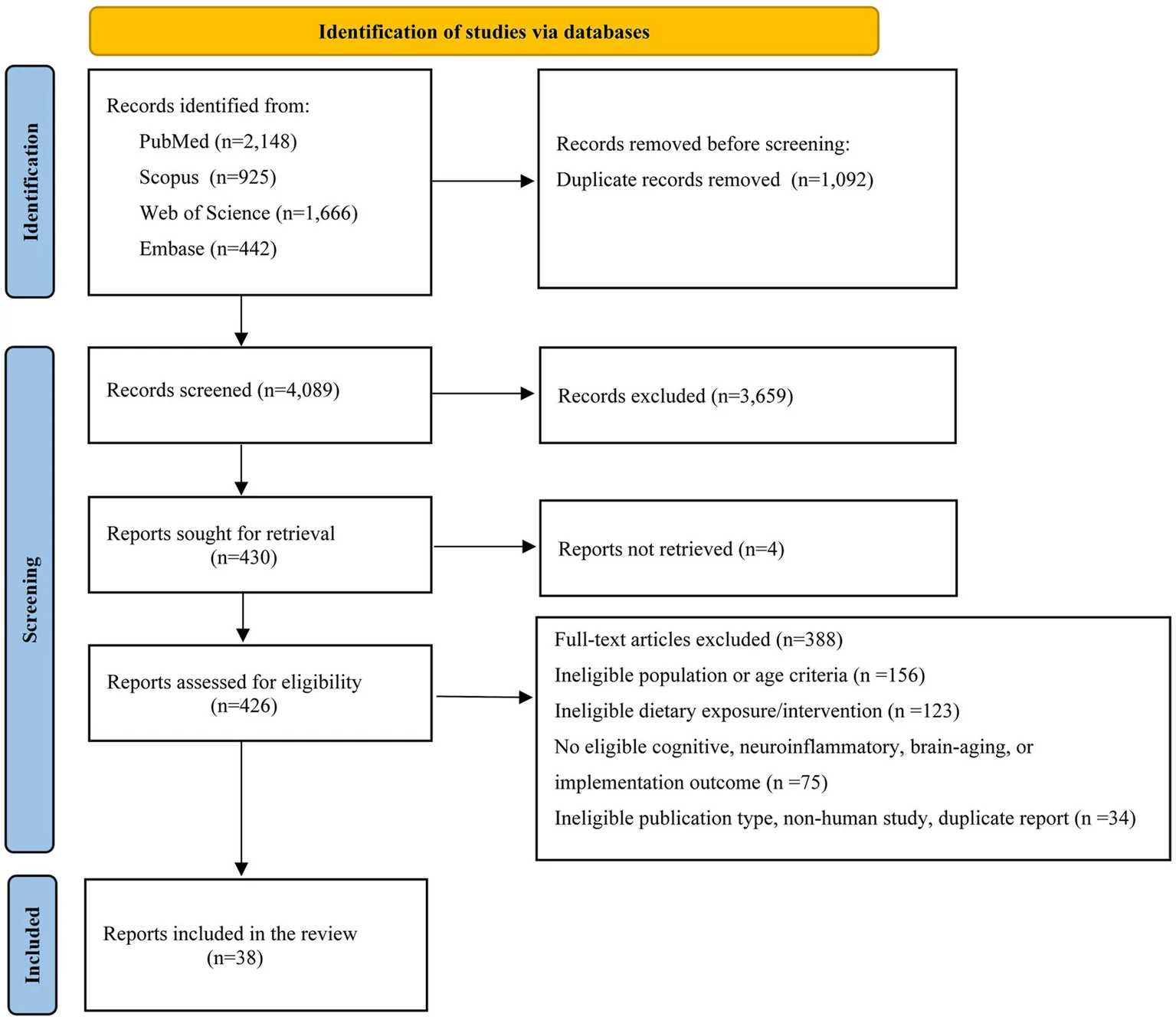
Preferred Reporting Items for Systematic Reviews and Meta-Analyses Extension for Scoping Reviews (PRISMA-ScR) flow diagram of study selection. Records identified, screened, assessed for eligibility, excluded with principal reasons, and included in the final evidence set.

The database searches identified 5,181 records, comprising 2,148 from PubMed, 925 from Scopus, 1,666 from Web of Science Core Collection, and 442 from Embase. After 1,092 duplicates were removed, 4,089 records underwent title and abstract screening, of which 3,659 were excluded. The full texts of 430 reports were sought, and four could not be retrieved. Of the 426 reports assessed for eligibility, 388 were excluded because they did not meet the population or age criteria (*n* = 156), did not evaluate an eligible dietary exposure or intervention (*n* = 123), did not report an eligible cognitive, neuroinflammatory, brain-aging, or implementation outcome (*n* = 75), or represented an ineligible publication type, non-human study, or duplicate report (*n* = 34). Thirty-eight reports were included in the final evidence set. The study-selection process is shown in Figure 1.

### Characteristics of the included evidence

The 38 included reports were published between 2006 and 2026; 20 reports (52.6%) were published from 2021 onward, including 16 (42.1%) published between 2023 and 2026. Sixteen reports were conducted in the United States and six in the United Kingdom (UK), while one combined cohorts from the UK and the United States and two included participants from several European countries. The remaining reports originated from Germany, Greece, France, the Netherlands, Spain, Sweden, China, Taiwan, and Australia. Twenty-seven reports (71.1%) were prospective or longitudinal observational analyses, seven (18.4%) were randomized dietary-intervention reports or secondary analyses of randomized interventions, three (7.9%) were cross-sectional biomarker or neuroimaging studies, and one (2.6%) was a *post hoc* observational analysis of randomized trial cohorts. The seven randomized reports represented five intervention programs because two publications were derived from the New Dietary Strategies Addressing the Specific Needs of the Elderly Population for Healthy Aging in Europe (NU-AGE) project and two from the Prevencion con Dieta Mediterranea (PREDIMED) trial. Analytic sample sizes ranged from 20 to 166,377 participants, while one report additionally included a meta-analysis of 224,049 participants. Reported mean ages ranged from 55.9 years at baseline in a cohort followed into later life to 84.1 years in a clinical-pathologic cohort. Observational follow-up generally ranged from approximately 3 to 22.3 years, with annual cognitive assessments extending for up to 23 years before death in one study; intervention follow-up ranged from 48 weeks to 6.5 years. Most reports included community-dwelling adults who were cognitively unimpaired or free of dementia at baseline, although selected samples were enriched for cardiometabolic disease, cardiovascular risk, overweight or obesity with metabolic syndrome, age-related macular degeneration, family history of dementia, subjective cognitive decline, or MCI. Reports derived from the same cohort or intervention, including the Chicago Health and Aging Project, Women’s Health Initiative Memory Study, Three-City Study, Memory and Aging Project, UK Biobank, NU-AGE, and PREDIMED, were linked at the study-family level rather than treated as independent replications. Detailed study characteristics are provided in Tables 1, 2 and Supplementary Table S2.

**Table 1:** Overview of the included evidence by study design (*N* = 38).

Study-design category	Reports, *n*	Typical design features	Principal evidence domains	Follow-up or assessment
Prospective or longitudinal observational analyses	27	Population-based and clinical cohorts, nested biomarker cohorts, longitudinal clinical–pathologic cohorts, and cohort analyses accompanied by meta-analysis	Repeated cognition, incident MCI/dementia, MRI or CSF markers, inflammatory proteins, microbiome, and neuropathology-adjusted cognition	Mostly 3–22.3 years
Randomized dietary-intervention reports or secondary randomized analyses	7	Multicentre randomized trials, site-specific cognitive substudies of a parent trial, feasibility/effectiveness trial, pilot randomized trial, and secondary microbiome analysis of a randomized intervention	Global and domain cognition, MRI, dietary adherence, feasibility, microbiome, and inflammatory markers	48 weeks–6.5 years
Cross-sectional biomarker or imaging studies	3	Multimodal biomarker study, observational neuroimaging study, and exploratory MRS study	Cognition, CSF amyloid/tau, regional brain volumes, systemic inflammation, barrier markers, and neuroinflammatory metabolites	Single assessment
*Post hoc* observational analysis of randomized trial cohorts	1	Observational dietary analysis within AREDS and AREDS2 cohorts	Cognitive impairment and longitudinal cognitive-test performance	Approximately 5–10 years

MCI, mild cognitive impairment; MRI, magnetic resonance imaging; CSF, cerebrospinal fluid; MRS, magnetic resonance spectroscopy. AREDS, Age-Related Eye Disease Study; AREDS2, Age-Related Eye Disease Study 2. Study-design categories were assigned once per report and are mutually exclusive.

**Table 2:** Primary dietary exposures represented in the included reports (*N* = 38).

Primary exposure category	Reports, *n*	Operationalization in included reports	Main evidence domains
Mediterranean or Mediterranean-style dietary patterns	20	MeDi or aMED scores, MEDAS, Mediterranean Pyramid scores, NU-AGE diet, and country-adapted Mediterranean scores	Global and domain cognition, dementia, MRI/DTI, CSF biomarkers, microbiome, and dietary intervention outcomes
MIND diet and MIND variants	10	Original MIND score, modified MIND score, and MIND-NL	Cognitive decline, incident impairment/dementia, cognitive resilience, systemic inflammatory markers, MRI, and randomized intervention outcomes
DII or energy-adjusted DII	6	Literature-derived inflammatory-potential scores calculated from available dietary parameters, with or without energy adjustment	Cognitive decline, MCI/dementia, MRI markers, systemic inflammatory proteins, and biomarker mediation
Empirical dietary inflammatory index/pattern	1	Food-based empirical inflammatory score	Memory performance and effect modification by periodontitis and *Helicobacter pylori* seropositivity
Combined DASH and Mediterranean dietary scores	1	Rank-based DASH and Mediterranean scores assessed in the same prospective cohort	Long-term global cognitive level

Primary exposure categories were assigned once per report and sum to 38. The report evaluating both DASH and Mediterranean scores was retained as a separate combined category. aMED, alternative Mediterranean diet; DASH, Dietary Approaches to Stop Hypertension; DII, Dietary Inflammatory Index; DTI, diffusion tensor imaging; MEDAS, Mediterranean Diet Adherence Screener; MeDi, Mediterranean diet score; MIND, Mediterranean–DASH Intervention for Neurodegenerative Delay; CSF, cerebrospinal fluid; MIND-NL, Netherlands-adapted MIND score; MRI, magnetic resonance imaging; NU-AGE, New Dietary Strategies Addressing the Specific Needs of the Elderly Population for Healthy Aging in Europe.

### Dietary exposures and outcome measurement

**Figure 2:**
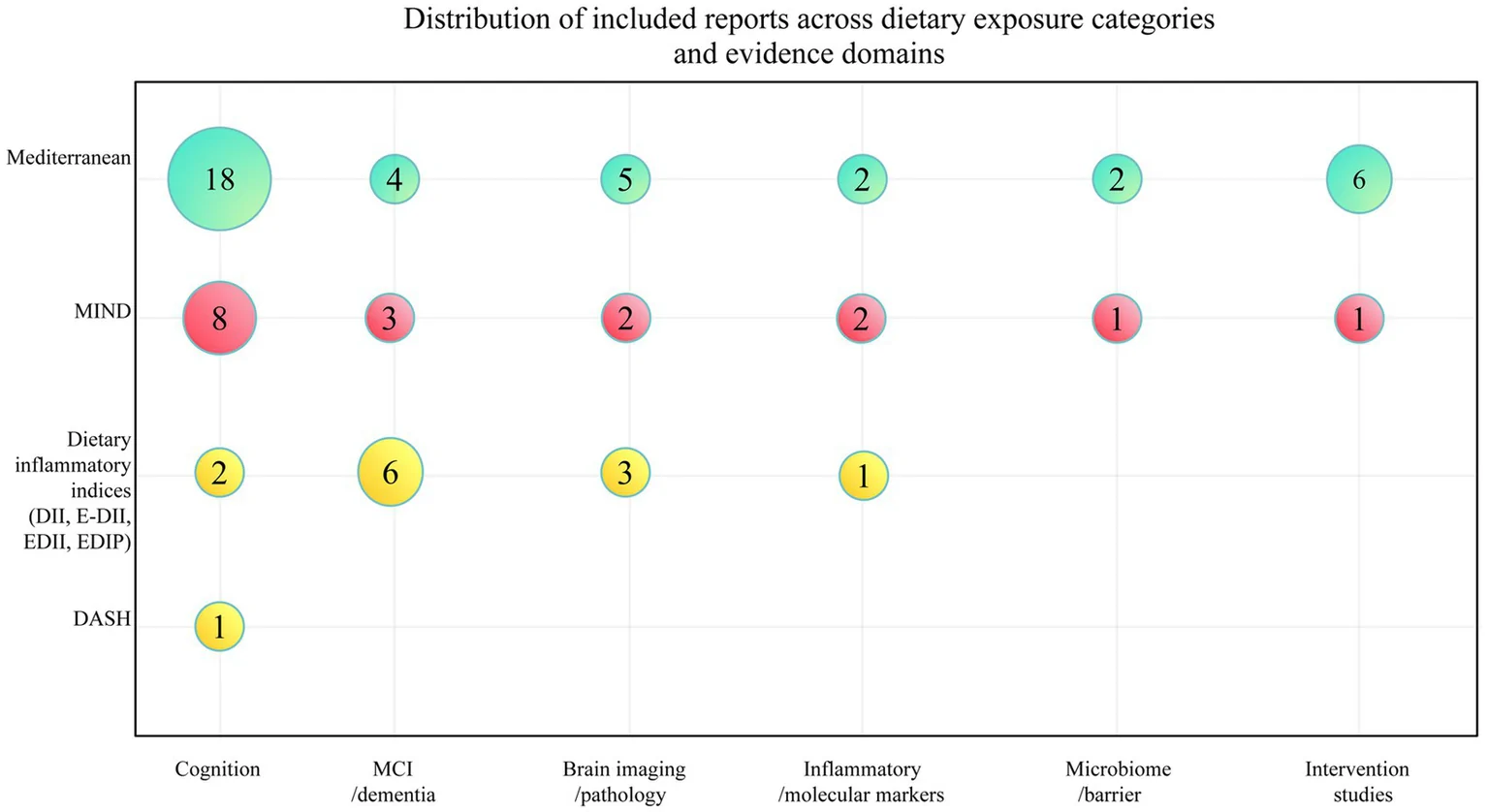
Distribution of included reports across dietary exposure categories and evidence domains. Values and bubble sizes represent the number of reports contributing to each non-mutually exclusive evidence domain. DASH, Dietary Approaches to Stop Hypertension; DII, Dietary Inflammatory Index; E-DII, energy-adjusted Dietary Inflammatory Index; EDII, Empirical Dietary Inflammatory Index; EDIP, Empirical Dietary Inflammatory Pattern; MCI, mild cognitive impairment; MIND, Mediterranean–DASH Intervention for Neurodegenerative Delay.

Mediterranean or Mediterranean-style dietary patterns were the primary exposure in 20 reports, MIND diets or locally modified MIND scores in 10, and literature-derived DII or E-DII scores in six. One report evaluated an EDII, and one assessed DASH and Mediterranean scores concurrently. Mediterranean exposures included traditional and alternative Mediterranean scores, the Mediterranean Diet Adherence Screener (MEDAS), Mediterranean Pyramid scores, country-adapted scores, and the NU-AGE intervention. MIND exposures included the original 0–15 score, a modified 0–12 score, and a Netherlands-adapted MIND score. DII calculations were based on between 29 and 45 available dietary parameters in reports that specified component availability. A food-frequency questionnaire was used as the principal dietary instrument or as part of the assessment in 26 reports; other studies used repeated 24-h recalls, 7-day food records or diaries, MEDAS-type screeners, or combinations of these methods. Exposures were modeled continuously or as study-specific tertiles, quartiles, quintiles, or adherence categories, and no common threshold defined high adherence or low dietary inflammatory potential. Cognitive outcomes included global and domain-specific performance, longitudinal cognitive decline, cognitive impairment, MCI, all-cause dementia, Alzheimer disease, and cognition unexplained by measured neuropathology. Biological outcomes included structural and diffusion MRI, CSF amyloid and tau markers, circulating inflammatory and immune markers, plasma proteomics, gut-microbiome profiles, intestinal-barrier markers, magnetic resonance spectroscopy metabolites, and postmortem neuropathology. Detailed dietary components and inflammatory characteristics are summarized in Supplementary Table S3. Figure 2 presents the non-mutually exclusive distribution of reports across exposure and evidence domains; detailed instruments and outcome definitions are reported in Supplementary Table S4.

### Clinical cognitive, MCI, and dementia outcomes

Findings from nonrandomized Mediterranean-pattern studies varied across cohorts and outcome definitions. In the Washington Heights–Inwood Columbia Aging Project, each 1-point increase in the Mediterranean diet score was associated with a lower incidence of Alzheimer disease (hazard ratio [HR] = 0.91, 95% confidence interval [CI], 0.83–0.98), and the highest versus lowest adherence tertile was associated with an HR of 0.60 (95% CI 0.42–0.87) (24). In the Chicago Health and Aging Project, each additional Mediterranean-diet score point was associated with a slower rate of global cognitive decline (*β* = 0.0014, *p* = 0.0004) (25). In UK Biobank, the highest versus lowest adherence category based on a continuous MEDAS-derived score was associated with lower dementia incidence (HR = 0.77, 95% CI 0.65–0.91), whereas the estimate based on the Mediterranean Pyramid score included the null value (HR = 0.86, 95% CI 0.73–1.02) (26). Higher MEDAS adherence was also associated with slower global cognitive decline in Prevención con Dieta Mediterránea-Plus (11). Féart et al. (27) reported less progression in Mini-Mental State Examination errors with higher adherence but no association with incident dementia (HR = 1.12, 95% CI 0.60–2.10). Keenan et al. (28) reported lower odds of cognitive impairment across the Age-Related Eye Disease Study and Age-Related Eye Disease Study 2, with odds ratios ranging from 0.36 to 0.56 for the highest versus lowest adherence categories, but no overall difference in the rate of cognitive decline. In the Cache County Study, the highest versus lowest Mediterranean-adherence quintile differed by 0.94 Modified Mini-Mental State Examination points, but a distinct difference in longitudinal slope was not demonstrated (29). Samieri et al. (30) and Vercambre et al. (31) found no overall association with global-cognition or verbal-memory trajectories, Titova et al. (32) reported no statistically significant association of the overall score with cognition or brain volume, and Gardener et al. (33) reported an association with executive function primarily among carriers of the apolipoprotein E ε4 allele.

Across the nine nonrandomized MIND reports, greater adherence was associated in some analyses with slower cognitive decline, lower incident cognitive impairment or dementia, more favorable inflammatory profiles, or greater cognitive resilience. However, the magnitude and consistency of these associations varied across populations, outcome definitions, covariate adjustment, and follow-up duration. One exploratory cross-sectional study reported interaction effects without an independent main association. Across three cohorts, each 3-point higher MIND score was associated with lower all-cause dementia incidence (pooled HR = 0.83, 95% CI 0.72–0.95) (34). In the Rotterdam Study, each 1-standard-deviation higher score was associated with lower dementia incidence during the first 7 years in both cohorts (HR = 0.85, 95% CI 0.74–0.98, and HR = 0.76, 95% CI 0.66–0.87), although the associations attenuated over longer follow-up (35). In the Memory and Aging Project, each additional score point was associated with a slower rate of global cognitive decline (*β* = 0.0092, *p* < 0.0001) (36). Greater adherence was also associated with slower cognitive decline in the Chicago Health and Aging Project and the Reasons for Geographic and Racial Differences in Stroke study, although estimates differed after adjustment for vascular and lifestyle factors and across sex and racial groups (37, 38). Huang et al. (39) reported a higher global cognitive level with greater adherence, while the meta-analytic estimate for annual decline included the null value. Higher MIND adherence was additionally associated with cognition unexplained by measured neuropathology and with global cognition in a prospective biomarker analysis (10, 40). In contrast, adherence to the Netherlands-adapted MIND score was not independently associated with cognition or spectroscopy-derived neuroinflammatory metabolites in the cross-sectional analysis by Remie et al. (12).

Across the six reports evaluating literature-derived DII or E-DII scores, more pro-inflammatory values were associated in some analyses with adverse cognitive, dementia, neuroimaging, or molecular outcomes. However, the outcome definitions, exposure contrasts, analytical approaches, and effect estimates were heterogeneous. Among reports presenting dementia incidence per DII unit, HRs ranged from 1.046 to 1.21, while comparisons of the most versus least pro-inflammatory categories produced HRs of approximately 1.25 to 3.00 (41–44). In adults with cardiometabolic disease, an anti-inflammatory rather than pro-inflammatory dietary profile was associated with lower dementia incidence (HR = 0.69, 95% CI 0.55–0.88) (8). The nested Women’s Health Initiative proteomic analysis identified inflammatory proteins associated with the E-DII score and subsequent cognitive impairment (9). In the single study of an empirical food-based inflammatory score, each 1-unit higher score was associated with poorer immediate free recall (*β* = −0.61, 95% CI − 1.02 to −0.21) and delayed free recall (*β* = −0.64, 95% CI − 1.06 to −0.22) (45). The only study assessing DASH and Mediterranean scores concurrently reported that the highest versus lowest DASH-adherence quintile differed by 0.97 Modified Mini-Mental State Examination points, while the difference in cognitive slope was not statistically significant (29). No included report tested DII or E-DII reduction as a randomized intervention target, and no dedicated randomized DASH trial was identified.

Seven reports arose from five randomized dietary-intervention programs. In the 3-year MIND trial involving 604 cognitively unimpaired older adults, global cognition improved in both the MIND and active-control groups, with no significant adjusted between-group difference at 3 years (mean difference = 0.035 standardized units, 95% CI -0.022 to 0.092; *p* = 0.23); no statistically significant between-group differences were observed in the four cognitive-domain scores or MRI outcomes (13). Two cognitive reports from the PREDIMED study family were also included. In the Barcelona-North substudy, 447 randomized participants completed baseline testing and 334 completed follow-up after a median of 4.1 years; selected memory, frontal, and global composite changes favored one of the Mediterranean-diet arms over control, although attrition, selected-substudy participation, and multiple outcomes limit interpretation (46). In PREDIMED-NAVARRA, 522 participants underwent blinded cognitive assessment after approximately 6.5 years, and adjusted end-of-follow-up Mini-Mental State Examination and Clock Drawing Test scores favored the Mediterranean-diet groups; the absence of baseline cognitive assessment prevented estimation of within-person cognitive change (47). Because both reports arose from the same parent trial, they were treated as one study family rather than independent confirmations. The NU-AGE trial randomized 1,279 independently living older adults and found no significant intention-to-treat differences in global cognition or five cognitive domains. In post-randomization adherence analyses, high versus low adherence was associated with global cognition (beta = 0.20, 95% CI 0.004–0.39) and episodic memory (beta = 0.15, 95% CI 0.02–0.28) (48). A separate analysis of 612 NU-AGE participants with paired microbiome data related diet-responsive taxa to selected cognitive and inflammatory measures but did not provide a separate randomized cognitive-effect estimate (49). The Mediterranean Diet, Exercise and Dementia Risk in UK Adults trial randomized 104 participants and reported between-group differences at 24 weeks in general cognition (0.22 standardized units, 95% CI 0.05–0.35) and memory (0.31, 95% CI 0.10–0.51); these differences were not maintained at 48 weeks (50). The Mediterranean diet and lifestyle education intervention randomized 20 participants, of whom 10 completed follow-up; recruitment difficulties, 50% attrition, and incomplete cognitive data precluded a stable or interpretable between-group estimate (51). Overall, randomized findings were mixed and constrained by selected substudies, attrition, endpoint-only assessment in one report, active comparators, exposure overlap, and limited power. The clinical findings are summarized in Table 3.

**Table 3:** Clinical evidence linking whole-diet patterns and dietary inflammatory indices with cognitive aging.

Dietary exposure	Evidence base	Principal outcomes	Integrated interpretation
Mediterranean and Mediterranean-style diets	20 primary-exposure reports plus 1 cohort evaluating DASH and Mediterranean scores; includes 6 randomized-intervention reports	Global and domain cognition, cognitive decline, incident dementia, MRI/DTI and CSF markers, and microbiome-related outcomes	Most prospective cohorts reported better cognitive level, slower decline, lower dementia risk, or more favorable brain markers, but several cohorts were null. Randomized evidence was mixed: two PREDIMED cognitive substudies reported favorable results but were limited by selected-substudy participation, attrition, and endpoint-only assessment in one report; NU-AGE showed no significant intention-to-treat cognitive effect; MedEx-UK reported short-term improvement that was not maintained; and THINK-MED was underpowered. Causal inference remains limited.
MIND diet and variants	10 reports, including one 604-participant, 3-year randomized trial	Global and domain cognition, incident cognitive impairment/dementia, cognitive resilience, inflammatory markers, and MRI outcomes	Observational cohorts generally favored slower decline or lower dementia risk, although associations varied by sex, race, and duration of follow-up. The randomized trial found no specific advantage over an active control for cognition or MRI outcomes, so observational protection has not been confirmed as a specific causal effect.
DASH diet	One prospective cohort that evaluated DASH and Mediterranean scores concurrently	Long-term global cognitive level and change	Higher DASH adherence was associated with an approximately 1-point higher 3MS score maintained over 11 years, but a differential rate of decline was not clearly demonstrated. No dedicated dementia-prevention trial was identified among the included reports.
DII and energy-adjusted DII	Six prospective observational reports	Cognitive decline, incident MCI/dementia, MRI markers, inflammatory proteomics, and biomarker mediation	More pro-inflammatory scores were generally associated with cognitive impairment or dementia and with adverse brain-imaging or inflammatory profiles. The evidence supports an association, but no randomized trial has tested reduction of dietary inflammatory potential as the intervention target.
Empirical inflammatory dietary patterns	One prospective community cohort	Immediate and delayed memory, with oral-health and infection-related effect modification	A more pro-inflammatory empirical score was associated with poorer memory. This evidence is emerging, culturally specific, and not independently replicated within the included set.

Overall, observational evidence predominantly favored healthier cognitive aging, whereas randomized evidence remained limited, mixed, or null. The findings support adjunctive risk-reduction counseling rather than a claim that diet alone prevents dementia. 3MS, Modified Mini-Mental State Examination; CSF, cerebrospinal fluid; DII, Dietary Inflammatory Index; DTI, diffusion tensor imaging; MCI, mild cognitive impairment; MIND, Mediterranean–DASH Intervention for Neurodegenerative Delay; MRI, magnetic resonance imaging.

### Directly measured biological pathways

Fourteen reports included directly measured inflammatory, molecular, microbiome, neuroimaging, CSF, spectroscopy, or neuropathologic outcomes. Fei et al. (10) measured 18 circulating inflammatory and immune markers and reported that cystatin C, soluble tumor necrosis factor receptor 1, interleukin-6, white blood cells, and interleukin-10 jointly mediated 13.3% of the reported MIND–cognition association. Duggan et al. (9) identified 55 plasma proteins associated with the E-DII score; seven proteins—C-X-C motif chemokine ligand 10, C-C motif chemokine ligand 3, hepatocyte growth factor, osteoprotegerin, CUB domain-containing protein 1, nuclear factor of activated T cells 3, and integrin subunit alpha 11—were also associated with subsequent cognitive impairment, and selected findings were partially replicated in external datasets. In NU-AGE, diet-responsive microbial taxa were associated with selected memory and constructional-praxis scores and with lower C-reactive protein and interleukin-17 concentrations (49). In the Prevención con Dieta Mediterránea-Plus study, a Mediterranean-diet microbial signature was independently associated with slower global cognitive decline and preserved executive function (11). Remie et al. (12) found that adherence to the Netherlands-adapted MIND score modified the associations of systemic inflammation and intestinal-barrier permeability with spectroscopy-derived neuroinflammatory metabolites, as well as the association between systemic inflammation and cognition, but was not independently associated with these outcomes.

**Figure 3:**
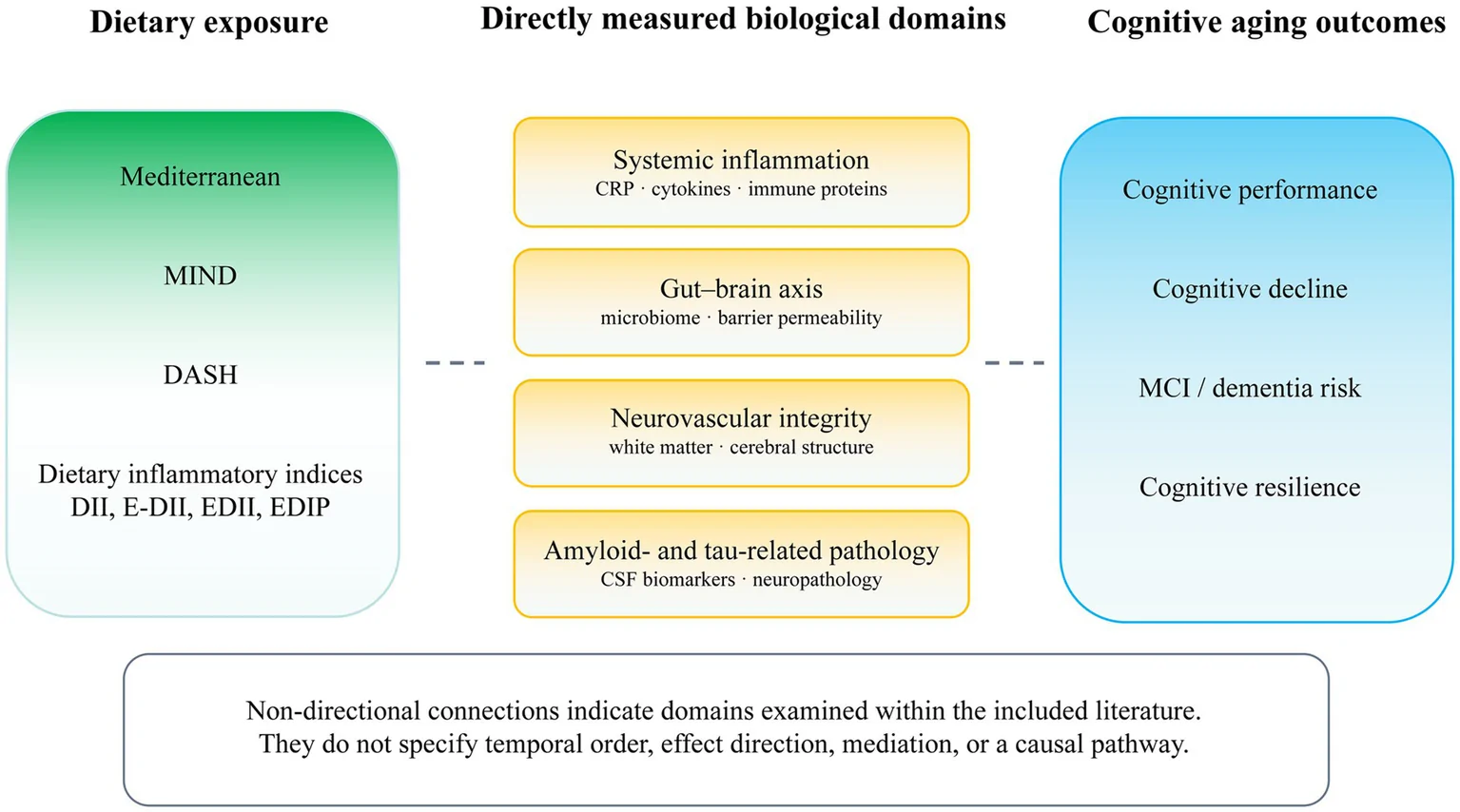
Associative map of dietary exposures, directly measured biological domains, and cognitive-aging outcomes. Non-directional connections indicate only that the linked domains were examined within the included literature; they do not specify temporal order, direction of effect, mediation, or a causal diet-mechanism-cognition pathway. CRP, C-reactive protein; CSF, cerebrospinal fluid; DASH, Dietary Approaches to Stop Hypertension; DII, Dietary Inflammatory Index; E-DII, energy-adjusted Dietary Inflammatory Index; EDII, empirical Dietary Inflammatory Index; MCI, mild cognitive impairment; MIND, Mediterranean-DASH Intervention for Neurodegenerative Delay; MRI, magnetic resonance imaging; EDIP, Empirical Dietary Inflammatory Pattern.

Structural and molecular brain-aging findings differed according to the measured region and modality. Higher Mediterranean adherence was associated with higher CSF amyloid-β42/40, lower phosphorylated tau-181, larger mediotemporal gray-matter volume, and better memory in the German Center for Neurodegenerative Diseases Longitudinal Cognitive Impairment and Dementia Study; the measured CSF and imaging variables statistically accounted for approximately 40% of the diet–memory association (52). Greater adherence was also associated with less total brain-volume loss over 3 years, larger dentate-gyrus volume, or preserved white-matter microstructure in selected studies, whereas associations with gray-matter volume, cortical thickness, white-matter hyperintensity burden, or the overall Mediterranean score were not consistently detected (32, 53–55). In UK Biobank, lower dietary inflammatory potential was associated with larger gray-matter volume and lower white-matter hyperintensity burden among participants with cardiometabolic disease, while each 1-unit higher DII was associated with 73.30 mm^3^ greater white-matter hyperintensity volume and lower hippocampal gray-matter volume in a separate analysis (8, 43). Higher MIND adherence was associated with a higher cognitive level and slower cognitive decline after adjustment for postmortem Alzheimer, vascular, Lewy-body, transactive response deoxyribonucleic acid-binding protein 43 kDa, hippocampal-sclerosis, and other neuropathologies (40). The randomized MIND trial found no statistically significant between-group differences in MRI-derived brain-volume changes (13). No included report directly quantified microglial activation as the principal mediator of a diet–cognition relationship, and no report evaluated a prespecified oxidative-stress biomarker as a formal mediator between a whole-diet exposure and subsequent cognition. The measured pathways are summarized in Figure 3, Table 4, and Supplementary Table S5.

**Table 4:** Biological pathways and intermediate markers examined in the included reports.

Measured pathway or domain	Directly measured evidence	Relevance to cognitive aging	Measurement characteristics and evidence limitations
Systemic inflammatory and immune signaling	Circulating CRP, IL-6, IL-10, IL-1RA, sTNFR-1, cystatin C, albumin, leukocyte measures, and high-dimensional plasma proteomics were examined. MIND-associated biomarkers jointly mediated 13.3% of the diet–cognition association in HRS; NU-AGE diet-responsive taxa were associated with lower CRP and IL-17.	Lower systemic inflammatory signaling and selected immune proteins were associated with better cognitive trajectories or lower subsequent cognitive-impairment risk.	Most findings were observational or secondary analyses. Statistical mediation does not establish causal biological mediation, and multiple-testing concerns remain for proteomic analyses.
Gut microbiome and intestinal-barrier pathways	NU-AGE assessed shotgun metagenomics and inferred metabolites; PREDIMED-Plus derived a Mediterranean-diet microbial signature; MIND-NL examined barrier-permeability markers and MRS neuroinflammatory metabolites.	Diet-responsive taxa or microbial signatures were associated with memory, executive function, frailty, inflammatory markers, or slower cognitive decline.	Microbiome outcomes were high-dimensional and often secondary, subset-based, or cross-sectional. The included reports did not demonstrate definitive microbiome-mediated cognitive benefit.
Neurovascular integrity and structural brain aging	Structural MRI and DTI measured WMH, total and regional gray matter, hippocampal or dentate-gyrus volume, cortical thickness, and white-matter microstructure.	Lower dietary inflammatory potential or greater Mediterranean adherence was associated in some cohorts with lower WMH burden, larger gray-matter or hippocampal volumes, preserved white-matter integrity, or slower total brain-volume loss.	Imaging findings were not uniform and frequently came from selected imaging subsets. Residual confounding and healthy-volunteer selection limit causal interpretation.
Amyloid/tau and mediotemporal neurodegeneration	DELCODE measured CSF Aβ42/40, p-tau181, mediotemporal gray-matter volume, and memory. Higher Mediterranean adherence was associated with a more favorable biomarker profile, and measured biomarkers statistically accounted for approximately 40% of the diet–memory association.	The findings provide convergent molecular, structural, and cognitive evidence relevant to Alzheimer-type neurodegeneration.	The analysis was cross-sectional and based on a smaller CSF subset; temporal direction and causal mediation cannot be established.
Neuropathology-adjusted cognitive resilience	The Memory and Aging Project combined annual cognition with postmortem assessment of Alzheimer, vascular, Lewy-body, TDP-43, hippocampal-sclerosis, and other pathologies.	Higher MIND adherence was associated with a higher cognitive level and slower decline after accounting for measured neuropathologies.	Cognitive resilience is a downstream phenotype rather than a directly measured biological mechanism. The findings do not demonstrate that diet reduced neuropathologic burden.

Major evidence gaps were direct measurement of microglial activation, prespecified oxidative-stress mediators, and formal causal mediation. Aβ, amyloid-β; CRP, C-reactive protein; CSF, cerebrospinal fluid; DTI, diffusion tensor imaging; IL, interleukin; MRI, magnetic resonance imaging; MRS, magnetic resonance spectroscopy; p-tau, phosphorylated tau; sTNFR-1, soluble tumor necrosis factor receptor 1; WMH, white-matter hyperintensity; TDP-43, transactive response DNA-binding protein 43 kDa. Study-family abbreviations are defined in the abbreviations section or main text.

### Methodological characteristics

Dietary exposure was predominantly measured through self-report, and many observational reports relied on a single baseline assessment. Repeated food-frequency questionnaires, multiple 24-h recalls, or repeated food records were available in a smaller subset. Dietary scores differed in component composition, scoring range, cut points, energy adjustment, and treatment as continuous or categorical variables. Cognitive ascertainment ranged from brief screening and telephone instruments to multidomain neuropsychological batteries, longitudinal composite scores, expert-adjudicated MCI or dementia, linked diagnostic records, and postmortem cognition–pathology models. MRI, CSF, proteomic, microbiome, and spectroscopy analyses were generally conducted in smaller or selected subsets of larger cohorts. High-dimensional proteomic and microbiome studies applied multiple-marker or false-discovery procedures; partial external replication of selected molecular findings was reported by Duggan et al. (9). Ballarini et al. (52), Karstens et al. (53), and Remie et al. (12) measured diet, biomarkers, and cognition cross-sectionally, whereas Fei et al. (10) temporally ordered dietary assessment, subsequent biomarker measurement, and repeated cognition. The mapped methodological characteristics are reported in Supplementary Table S6.

The design-specific appraisal materially qualified the synthesis. Observational evidence was interpreted in light of self-reported exposure, residual confounding, reverse causation, selective survival, and healthy-adherer bias. Randomized evidence was interpreted in light of intervention contrast, adherence, attrition, post-randomization analyses, and selected cognitive substudies; the PREDIMED-NAVARRA report also lacked baseline cognitive assessment. Mechanistic subsets were interpreted in light of temporal ambiguity, multiplicity, selected sampling, and limited external validation. These domains were not converted into summary scores, but they prevented report counts or statistical significance from being treated as equivalent evidence of maturity or consistency.

### Implementation evidence and nursing implications

**Figure 4:**
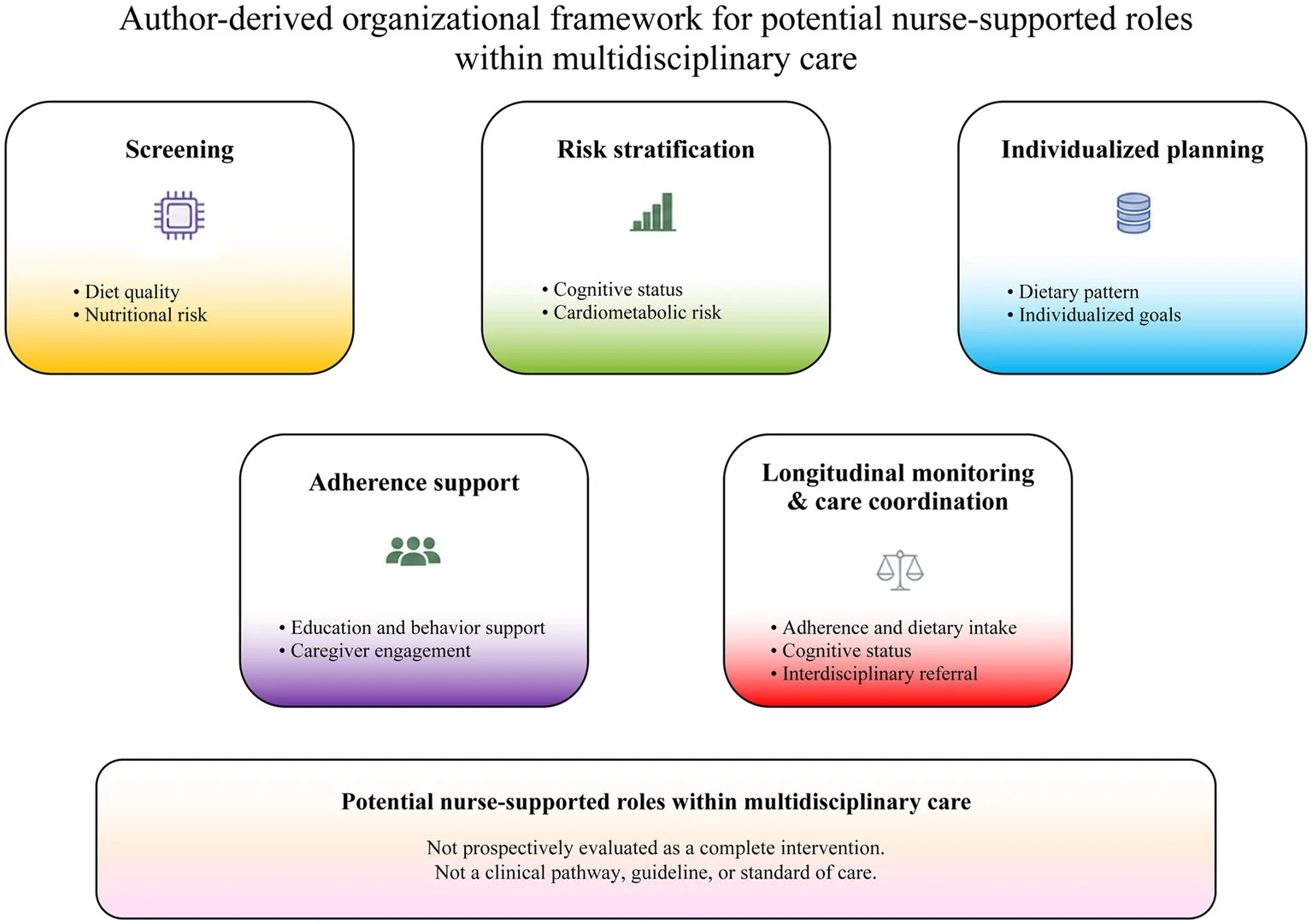
Author-derived organizational framework for potential nurse-supported roles within multidisciplinary care. The framework organizes screening, risk stratification, individualized planning, adherence support, and longitudinal monitoring and care coordination. It has not been prospectively evaluated as a complete intervention and is not a clinical pathway, guideline, or standard of care.

Implementation-related data were concentrated in the seven randomized reports and mainly covered recruitment, retention, dietary adherence, intervention contact, feasibility, and maintenance of dietary change. Across these reports, constraints included attenuation of adherence after active support, recruitment and retention difficulties, incomplete cognitive follow-up, reliance on post-randomization adherence analyses, selected cognitive substudies, and limited exposure separation under an active comparator (13, 46–48, 50, 51). No included report prospectively evaluated a complete nurse-led or nurse-supported dietary pathway for cognitive-risk reduction, routine integration into nursing documentation, prespecified referral thresholds, interprofessional follow-up ownership, economic outcomes, or real-world service deployment. Caregiver participation, acceptability, workflow effects, and equity-stratified implementation outcomes were not reported consistently. The recurring implementation and care-delivery elements were used only to organize the author-derived framework shown in Figure 4 and Supplementary Tables S7A,B. The framework proposes potential nurse-supported roles within multidisciplinary care; it was not evaluated as a complete intervention, clinical pathway, guideline, or standard of care.

## Discussion

### Overall interpretation: the gap between observational evidence and randomized trials

This review does not support the conclusion that a specific “anti-inflammatory diet” prevents dementia. A more defensible interpretation is that the evidence has matured unevenly across study designs. Prospective observational studies provide the most consistent signal, directly measured human studies add biological plausibility, and randomized trials impose the strongest constraint on causal claims but remain limited and largely nonconfirmatory. We therefore interpret the literature as epidemiologically suggestive, biologically coherent, and clinically relevant, but not causally settled. This position is consistent with recent syntheses showing that observational associations are more coherent than the cognitive effects demonstrated in whole-diet intervention trials among older adults (7, 15, 16).

The observational–randomized gap should not be treated as a contest in which one design must be correct and the other mistaken. The two bodies of evidence estimate different exposure contrasts. Observational studies compare dietary patterns accumulated over years and embedded within broader patterns of education, physical activity, vascular-risk management, social support, and food access. Randomized trials ask whether a defined intervention produces an incremental benefit over a comparator during a limited period in selected volunteers. Observational estimates may better reflect cumulative exposure, but they remain vulnerable to residual confounding, healthy-adherer bias, selective survival, measurement error, and reverse causation. Trials reduce baseline confounding, yet the achievable contrast may be modest when control participants also receive counseling, improve their diet, or begin with relatively favorable eating patterns.

Null or attenuated trial findings should therefore be taken seriously rather than dismissed as methodological inconvenience. The MIND trial, NU-AGE, and the Mediterranean Diet, Exercise and Dementia Risk in UK Adults trial indicate that dietary behavior can improve without producing a durable between-group cognitive advantage under the tested conditions (13, 48, 50). Active comparators, practice effects, relatively healthy volunteers, limited follow-up, and intervention initiation after decades of vascular or neuropathological exposure may reduce detectable differences. Even so, these explanations do not convert a null trial into a positive one. The appropriate inference is that a specific additional cognitive benefit was not established in those settings. Post-randomization adherence analyses do not fully bridge this gap because grouping participants by achieved adherence weakens the protection afforded by random assignment. Highly adherent participants may differ in motivation, baseline health, cognition, or social support. Such analyses help determine whether behavioral separation occurred and can generate dose-related hypotheses, but they are not equivalent to intention-to-treat evidence. Overall, the divergence between designs places an important boundary around the field: healthier dietary patterns remain plausible components of cognitive-risk reduction, but the magnitude and causal independence of any diet-specific effect remain uncertain.

### Heterogeneity of dietary exposures and conditions of effect

The umbrella term “anti-inflammatory diet” conceals materially different exposure constructs. Mediterranean, MIND, and DASH scores describe adherence to predefined combinations of foods selected for their nutritional, vascular, metabolic, or brain-health relevance. The DII and E-DII estimate dietary inflammatory potential using literature-derived weights, whereas EDIP and related empirical indices derive food-group weights from associations with circulating inflammatory biomarkers (5, 6). These constructs overlap, but they do not measure a single interchangeable biological exposure. A higher MIND score is not a direct measure of inflammatory activity, and a lower DII score does not demonstrate that systemic or central inflammation changed in an individual participant.

Dietary scores are measurement architectures rather than fixed biological entities. Their meaning depends on the components available in the source instrument, energy adjustment, adaptations to the original score, cohort-specific cut points, and whether adherence is modeled continuously or categorically. Two studies may therefore use the same label while representing substantially different absolute diets. Cross-cultural transportability adds further uncertainty. A score developed in one food environment may perform differently in another, while access to recommended foods is shaped by income, health literacy, oral health, cooking facilities, household roles, and caregiver support. Apparent variation by sex, race, socioeconomic position, cardiometabolic status, or genotype may reflect genuine biological modification, but it may also reflect measurement performance and unequal opportunity to achieve the prescribed pattern (37, 38).

The likely effect of diet is also conditional rather than uniform. Timing matters: a pattern maintained from midlife is not biologically equivalent to an intervention introduced after substantial neurovascular or neurodegenerative injury has accumulated. Baseline diet quality determines the room for improvement, while cardiometabolic burden, genetic susceptibility, frailty, oral and swallowing function, and the ability to sustain behavioral change may modify the response. In our view, diet is best conceptualized as a long-horizon risk-modifying exposure whose effects are likely modest, cumulative, and context dependent. It has no single active ingredient, fixed dose, or inert placebo. The relevant exposure is not merely a score, but a sustained behavioral and material system operating across the life course.

### Maturity of the mechanistic evidence: from peripheral inflammation to cognitive resilience

The mechanistic literature has moved beyond speculation, but it has not established a complete diet–neuroinflammation–cognition pathway. Human studies have identified plausible links involving circulating inflammatory and immune markers, plasma proteins, gut-microbial ecology, intestinal-barrier function, neurovascular integrity, structural brain aging, CSF amyloid and tau markers, and cognition unexplained by measured neuropathology (9–11, 40, 49, 52). Convergence across these domains is meaningful, but the evidence resembles a mosaic of partially connected findings rather than a prospectively verified causal chain.

The central conceptual boundary is the distinction between peripheral inflammation and neuroinflammation. C-reactive protein, cytokines, leukocyte measures, soluble receptors, and circulating proteins indicate systemic immune activity, but they do not directly quantify microglial activation or region-specific immune signaling within the brain. Central immune responses are heterogeneous and may be protective, compensatory, or harmful depending on disease stage and cellular context (3, 4). These gaps are consequential: without direct central immune measures or temporally ordered testing across oxidative-stress and blood–brain barrier pathways, peripheral biomarker findings cannot be interpreted as evidence that a diet reduces neuroinflammation.

Causal mediation remains similarly uncertain. Observational mediation models require strong assumptions regarding unmeasured confounding, while cross-sectional CSF, spectroscopy, or imaging analyses cannot exclude the possibility that preclinical cognitive or functional change had already altered food selection and self-management. High-dimensional proteomic and microbiome studies add biological resolution but also introduce multiplicity, platform heterogeneity, subset selection, and a need for independent replication. A statistically associated mediator does not establish that modifying that mediator would alter cognitive outcomes. The distinction between pathology modification and cognitive resilience is equally important. Favorable amyloid, tau, white-matter, or regional-volume profiles are compatible with an effect on disease processes. Better cognition after accounting for measured neuropathology suggests that diet may instead modify the functional expression of pathology through vascular, synaptic, metabolic, or reserve-related processes (40, 52). In our interpretation, inflammation is likely to be one component of a broader network rather than the single bridge between diet and cognition. Evidence of systemic immune regulation, central pathology, and cognitive resilience should therefore be interpreted as related but distinct domains.

### Clinical translation: nutritional safety before the “anti-inflammatory” label

The clinical message should remain proportional to the evidence. Improving dietary quality is reasonable as part of multidomain cognitive-risk reduction because diet intersects with hypertension, diabetes, obesity, dyslipidemia, vascular health, physical activity, and other modifiable determinants of cognitive aging. It should not be presented as a stand-alone treatment or a proven method of preventing dementia. Contemporary prevention frameworks similarly emphasize interacting risk domains, and multidomain trials demonstrate the feasibility of combining nutritional guidance with exercise, cognitive training, and vascular-risk management without establishing that diet alone produced the benefit (2, 56).

Translation should be principle-based rather than cuisine-based. The relevant principles are improved overall dietary quality, greater intake of nutrient-dense plant foods, appropriate sources of unsaturated fat, and reduced reliance on highly processed foods. They should be adapted to local foods, cultural practices, affordability, comorbidity, oral health, swallowing ability, functional status, and personal preference. Requiring an older adult to reproduce a rigid Mediterranean menu is neither necessary nor realistic. A recommendation that is nutritionally sound but materially unattainable is not an effective intervention.

Nutritional safety is the principal boundary. For older adults with frailty, poor appetite, dysphagia, unintended weight loss, or established malnutrition, adequate energy, protein, hydration, and tolerable intake take precedence over maximizing an inflammatory or adherence score. Restrictive advice delivered under an “anti-inflammatory” label could worsen nutritional status. Current dementia nutrition guidance likewise prioritizes screening, individualized assessment, adequate intake, and sustained social and nursing support rather than nutritional interventions as treatments for cognitive impairment itself (17). Nutritional adequacy is therefore a prerequisite for dietary optimization, not a secondary caveat. The credible clinical message is that safe and achievable improvements in diet quality may contribute to broader risk reduction without displacing established assessment, treatment, or individualized nutrition care.

### Nursing implications and proposed translation framework

Potential nursing relevance arises from continuity of contact and knowledge of the patient’s functional and social context. Across prevention, chronic-disease management, rehabilitation, community follow-up, and caregiver support, nurses may help identify poor diet quality alongside malnutrition risk, oral or swallowing problems, cognitive and functional limitations, food insecurity, medication-related barriers, and reduced capacity to shop or prepare meals. Within multidisciplinary care, potential roles include screening, shared goal-setting, practical education, adherence support, monitoring, caregiver engagement, and referral coordination; these roles should not be interpreted as evidence for a tested nurse-led intervention.

The author-derived five-stage framework (screening, stratification, individualized planning, adherence support, and longitudinal monitoring and care coordination) is intended only to organize potential activities. It is not an established clinical pathway. Because no included study evaluated the complete framework, its effects on cognition or dementia incidence and its implications for workload, cost, referral capacity, acceptability, and professional accountability remain unknown. Prospective effectiveness-implementation studies should assess reach, adoption, fidelity, retention, referral completion, caregiver burden, nutritional adequacy, unintended weight loss, staff workload, economic consequences, and maintenance after active support ends.

Equity should be treated as an implementation outcome rather than an assumed benefit. Dietary advice is enacted within households and food systems, and its feasibility depends on cost, availability, transport, language, health literacy, oral function, cooking capacity, cultural meaning, and caregiver support. A pathway that works mainly for food-secure, highly educated, mobile, and well-supported participants could widen disparities while appearing effective on average. Equity-oriented implementation would therefore require culturally relevant food examples, assessment of affordability and food insecurity, flexible delivery, adequate referral capacity, and reporting of uptake and outcomes across socioeconomic, cultural, and functional groups. These are author-proposed implementation considerations rather than effects established by the included studies.

### Research priorities, strengths, and limitations

Future studies should move beyond asking whether a named diet is generally “good for the brain.” More informative questions concern who is most likely to benefit, at what stage of risk, through which pathway, with what exposure contrast, and under what delivery conditions. Trials require adequate power and longer follow-up, but duration alone will not correct weak exposure separation. Intervention and comparator diets should differ meaningfully, intake should be measured repeatedly, maintenance after active support should be documented, and intention-to-treat analysis should remain primary. Objective dietary biomarkers and technology-assisted assessment may help distinguish biological inefficacy from failure to create or sustain the intended contrast.

Outcome selection also requires greater discipline. A core set should include global and domain-specific cognition, functional outcomes, clinically adjudicated MCI or dementia where feasible, dietary adherence, and nutritional safety. Mechanistic substudies should use repeated, temporally ordered measures rather than isolated cross-sectional panels. Diet, systemic inflammation, microbiome and metabolic change, neurovascular or central markers, and cognition should be assessed in a sequence that permits credible mediation testing. High-dimensional analyses require prespecification, multiplicity control, and independent replication.

Implementation research should proceed alongside efficacy testing. Hybrid effectiveness–implementation designs could examine culturally adapted, nurse-supported programs while measuring clinical, nutritional, organizational, equity, and economic outcomes. When diet is combined with exercise, cognitive training, or vascular-risk management, factorial designs, separable components, or prespecified mediation analyses are needed to preserve interpretability. The Finnish Geriatric Intervention Study to Prevent Cognitive Impairment and Disability experience demonstrates the feasibility of multidomain prevention, but it does not identify which components are necessary, sufficient, or most scalable (56).

This review has several strengths. It preserved the distinction between food-pattern scores and inflammatory indices, separated observational associations from randomized effects, linked reports from the same cohort or intervention, and restricted mechanistic claims to pathways directly measured in humans. It also treated clinical and nursing implementation as a distinct evidence domain rather than assuming that epidemiological association automatically translates into practice. The structured mapping of cognitive, biological, and implementation evidence clarified where the literature is mature and where it remains inferential.

Several limitations constrain interpretation. No formal tool-based risk-of-bias classification or certainty-of-evidence rating was undertaken, consistent with the scoping-review design; the design-specific appraisal therefore qualifies but does not replace formal risk-of-bias assessment. Heterogeneity in dietary definitions, scoring systems, cognitive instruments, biological measures, follow-up, and effect metrics precluded quantitative pooling. Several reports arose from overlapping cohorts or trials, so report counts do not represent an equivalent number of independent replications. The evidence was concentrated in the United States and Europe and largely involved community-dwelling adults without dementia, limiting applicability to institutionalized, hospitalized, frail, and non-Western populations. English-language restriction may have excluded relevant evidence. The mechanism-related AND block used in Embase, Web of Science, and Scopus may have missed eligible diet-cognition reports without mechanistic indexing, and the asymmetric database strategies cannot be assumed to have equivalent sensitivity. Targeted re-screening added the two reviewer-identified PREDIMED reports, but it does not establish that no other eligible report was missed. Finally, the nursing framework was derived from available evidence and care functions; it has not been validated as an intervention, clinical pathway, guideline, or standard of care.

Accordingly, this review should be read as a map of evidence maturity rather than an estimate of treatment efficacy.

## Conclusion

Higher-quality Mediterranean- and MIND-oriented dietary patterns and lower dietary inflammatory potential are recurrently associated with more favorable cognitive aging, but the current evidence does not establish that any specific diet prevents dementia. Observational associations are more developed than randomized evidence, and human biomarker, microbiome, neuroimaging, CSF, and neuropathologic studies support only parts of the proposed biological pathway. Inflammation is therefore best viewed as one component of a broader vascular, metabolic, microbial, and neurodegenerative network rather than a fully demonstrated causal mechanism.

Dietary improvement can reasonably be incorporated into individualized, multidomain risk reduction, provided that cultural fit, affordability, functional capacity, and nutritional safety are addressed. For frail or malnourished older adults, adequate intake remains the priority. Nurses may contribute through screening, practical education, adherence support, monitoring, caregiver engagement, and care coordination, but the proposed framework requires prospective effectiveness and implementation testing. Progress will depend on better-contrasted trials, temporally ordered mechanistic studies, harmonized outcomes, and real-world evaluations of feasibility, equity, safety, cost, and sustained benefit.

## Data Availability

The raw data supporting the conclusions of this article will be made available by the authors, without undue reservation.
