# Supplementary material for: Anti-inflammatory dietary patterns and cognitive aging in older adults: a scoping review of mechanistic, clinical, and nursing implications
Source: Front Nutr. 2026 Sep 18;13:1952047. doi: 10.3389/fnut.2026.1952047 (PMC13589688; doi:10.3389/fnut.2026.1952047)
Supplement: Supplementary file 1 [file Table_1.docx]

Supplementary Table S1. Search strategies for database searches

| **Database** | **Search strategy** | **Records retrieved** |
| --- | --- | --- |
| PubMed | ( "anti-inflammatory diet*"[Title/Abstract] OR "Dietary Inflammatory Index"[Title/Abstract] OR DII[Title/Abstract] OR "inflammatory diet*"[Title/Abstract] OR "Mediterranean diet*"[Title/Abstract] OR "MIND diet*"[Title/Abstract] OR "Mediterranean-DASH Intervention for Neurodegenerative Delay"[Title/Abstract] OR "DASH diet*"[Title/Abstract] OR "plant-based diet*"[Title/Abstract] OR "dietary pattern*"[Title/Abstract] OR "healthy dietary pattern*"[Title/Abstract] OR omega-3[Title/Abstract] OR polyphenol*[Title/Abstract] OR flavonoid*[Title/Abstract] OR fiber[Title/Abstract] ) AND ( "cognitive decline"[Title/Abstract] OR "cognitive decline*"[Title/Abstract] OR "cognitive impairment"[Title/Abstract] OR "mild cognitive impairment"[Title/Abstract] OR MCI[Title/Abstract] OR dementia*[Title/Abstract] OR Alzheimer*[Title/Abstract] OR "cognitive aging"[Title/Abstract] OR "brain aging"[Title/Abstract] OR "cognitive frailty"[Title/Abstract] ) AND ( aging[Title/Abstract] OR ageing[Title/Abstract] OR elderly[Title/Abstract] OR "older adult*"[Title/Abstract] OR "older people"[Title/Abstract] ) | 2148 |
| Embase | ('anti inflammatory diet*':ti,ab OR 'dietary inflammatory index':ti,ab OR 'mediterranean diet'/exp OR 'mediterranean diet*':ti,ab OR 'mind diet':ti,ab OR 'dash diet':ti,ab OR 'plant based diet*':ti,ab OR 'dietary pattern*':ti,ab OR omega-3:ti,ab OR polyphenol*:ti,ab) AND ('cognitive decline'/exp OR 'cognitive impairment':ti,ab OR dementia:ti,ab OR Alzheimer*:ti,ab OR 'mild cognitive impairment':ti,ab OR 'cognitive frailty':ti,ab) AND (neuroinflammation:ti,ab OR inflammation:ti,ab OR inflammaging:ti,ab OR microglia*:ti,ab OR cytokine*:ti,ab OR oxidative stress:ti,ab OR microbiota:ti,ab OR 'gut brain axis':ti,ab) AND (aged:de OR aging:ti,ab OR elderly:ti,ab OR 'older adult*':ti,ab) | 442 |
| Web of Science Core Collection | TS=("anti-inflammatory diet*" OR "Dietary Inflammatory Index" OR DII OR "inflammatory diet*" OR "Mediterranean diet*" OR "MIND diet*" OR "Mediterranean-DASH" OR "DASH diet*" OR "plant-based diet*" OR "dietary pattern*" OR omega-3 OR polyphenol* OR flavonoid*) AND TS=("cognitive decline*" OR "cognitive impairment*" OR cognition OR dementia* OR Alzheimer* OR "mild cognitive impairment" OR "cognitive frailty") AND TS=(neuroinflammation OR inflammation OR inflammaging OR microglia* OR cytokine* OR oxidative stress OR "gut-brain axis" OR microbiota OR immunometabolism OR "blood brain barrier") AND TS=(aging OR ageing OR elderly OR "older adult*") | 1666 |
| Scopus | TITLE-ABS-KEY("anti-inflammatory diet*" OR "Dietary Inflammatory Index" OR DII OR "Mediterranean diet*" OR "MIND diet*" OR "DASH diet*" OR "plant-based diet*" OR "dietary pattern*" OR omega-3 OR polyphenol*) AND TITLE-ABS-KEY("cognitive decline*" OR "cognitive impairment*" OR dementia* OR Alzheimer* OR cognition OR "cognitive frailty") AND TITLE-ABS-KEY(neuroinflammation OR inflammation OR inflammaging OR microglia* OR cytokine* OR oxidative stress OR "gut-brain axis" OR microbiota OR immunometabolism) AND TITLE-ABS-KEY(aging OR elderly OR "older adult*") | 925 |

Supplementary Table S2. Characteristics of included reports (N=38)

| **Study** | **Country** | **Study design** | **Population** | **Sample size** | **Age** | **Dietary pattern** | **Dietary assessment** | **Follow-up** | **Cognitive outcome** | **Main findings** |
| --- | --- | --- | --- | --- | --- | --- | --- | --- | --- | --- |
| Agarwal et al. (2024) | United States | Prospective population-based cohort study (Chicago Health and Aging Project) | Black and White older adults | 5,259 | Mean 73.5±6.0 years | MIND diet | FFQ; 0–15 MIND score | Mean 7.8±4.6 years | Change in global cognition | Greater MIND adherence was associated with slower decline overall (P for trend=.0025). In White participants, tertile 2 β=.0121 (95% CI .0006–.0237) and tertile 3 β=.0146 (.0003–.0260); in Black participants, tertile 3 β=.0088 (.0003–.0172). Adjustment for vascular/lifestyle factors attenuated the Black-participant estimate. |
| Ballarini et al. (2021) | Germany | Cross-sectional multimodal biomarker study (DELCODE) | Cognitively normal adults, first-degree relatives of patients with AD, subjective cognitive decline, and MCI | 512; cerebrospinal-fluid subset 226 | Mean 69.5±5.9 years | Mediterranean diet | FFQ; Mediterranean-diet adherence score | Cross-sectional | Memory, mediotemporal gray-matter volume, CSF Aβ42/40, and phosphorylated tau | Higher Mediterranean-diet adherence was associated with better memory (β=.03±.02; P=.038), higher CSF Aβ42/40 (β=.003±.001; P=.008), lower p-tau181 (β=−1.96±.68; P=.004), and larger mediotemporal gray-matter volume (P<.05 familywise-error corrected). Biomarkers accounted for approximately 40% of the diet–memory association. |
| Barnes et al. (2023) | United States | Multicentre randomized controlled trial | Cognitively unimpaired older adults with family history of dementia, BMI>25, and suboptimal diet | 604 randomized (301 MIND; 303 control) | Mean 70.4±4.2 years | MIND diet with mild caloric restriction versus control diet with mild caloric restriction | 14-item FFQ-derived MIND score plus repeated dietary recalls and adherence monitoring | 3 years | Global cognition, four cognitive domains, and MRI brain outcomes | Global cognition improved in both groups, with no significant between-group difference at 3 years (mean difference=.035 standardized units, 95% CI −.022 to .092; P=.23). Four cognitive domains and MRI outcomes likewise did not differ materially. |
| Charisis et al. (2021) | Greece | Prospective population-based cohort study (HELIAD) | Community-dwelling older adults without dementia | 1,059 | Mean 73.1 years | Dietary Inflammatory Index (DII) | Detailed FFQ; DII based on 45 food parameters | Mean 3.05±0.85 years | Incident dementia | Each 1-unit higher DII was associated with incident dementia (HR=1.21, 95% CI 1.03–1.42; P=.023). Highest versus lowest DII tertile HR=3.0 (95% CI 1.2–7.3; P=.014; P for trend=.014). |
| Chen et al. (2023) | United Kingdom and United States | Prospective analyses of three cohorts plus meta-analysis | Whitehall II, Health and Retirement Study, and Framingham Offspring participants | 18,136 cohort participants; meta-analysis 224,049 participants | WII 62.2±6.0; HRS 66.5±10.4; FOS 64.2±9.1 years | MIND diet | Cohort-specific validated FFQs; 0–15 MIND score | 166,516 person-years across cohorts | Incident all-cause dementia | Across the three cohorts, each 3-point higher MIND score was associated with lower dementia risk (pooled HR=0.83, 95% CI 0.72–0.95; P for trend=.01). In the broader meta-analysis, highest versus lowest MIND tertile HR=0.83 (95% CI 0.76–0.90; I²=35%). |
| Chou et al. (2025) | Taiwan | Prospective community-based cohort study | Community-dwelling adults aged ≥65 years | 511 | ≥65 years | Empirical Dietary Inflammatory Index (EDII) | 44-item semiquantitative FFQ; food-based EDII | Baseline and three biennial follow-ups (approximately 6 years) | Global cognition, memory, attention, executive function, and verbal fluency | Each 1-unit higher EDII was associated with poorer immediate free recall (β=−0.61, 95% CI −1.02 to −0.21; P=.003) and delayed free recall (β=−0.64, 95% CI −1.06 to −0.22; P=.003). Associations were stronger in participants with periodontitis and Helicobacter pylori seropositivity; EDII×periodontitis interaction for memory P=.01. |
| Dove et al. (2024) | United Kingdom | Prospective cohort study (UK Biobank) | Dementia-free adults aged ≥60 years, stratified by cardiometabolic disease (CMD) | 84,342; brain-MRI subset 8,917 | Mean 64.1±2.9 years | Anti-inflammatory versus pro-inflammatory diet assessed by DII | Up to five Oxford WebQ 24-hour dietary assessments; DII calculated from 31 components | Median 12.4 years (maximum 15 years) | Incident dementia; gray-matter volume and white-matter hyperintensity (WMH) volume | Among participants with CMD, an anti-inflammatory versus pro-inflammatory diet was associated with lower dementia risk (HR=0.69, 95% CI 0.55–0.88; P=.003). CMD plus a pro-inflammatory diet had HR=2.38 (1.93–2.93), whereas CMD plus an anti-inflammatory diet had HR=1.65 (1.36–2.00), relative to CMD-free/anti-inflammatory participants. Anti-inflammatory diet was also associated with larger gray-matter and smaller WMH volumes. |
| Duggan et al. (2023) | United States | Prospective nested proteomic cohort (Women’s Health Initiative Memory Study) | Cognitively unimpaired older women at baseline | 1,528 | Mean 71.30±3.75 years | Energy-adjusted Dietary Inflammatory Index (E-DII) and Dietary Inflammatory Index (DII) | WHI FFQ based on the Block FFQ; E-DII and DII | Mean 13.55±5.20 years | Incident MCI or probable dementia and inflammatory/immune proteomic signatures | Fifty-five plasma proteins were associated with E-DII. Seven candidate proteins (CXCL10, CCL3, HGF, OPG, CDCP1, NFATC3, and ITGA11) were related to future cognitive impairment; several associations were replicated in ARIC/ESTHER and correlated with Aβ42/40, neurofilament light, or brain atrophy. |
| Fei et al. (2025) | United States | Prospective cohort and mediation analysis (Health and Retirement Study) | Community-dwelling middle-aged and older adults | 3,777 | Mean 65.3 years | MIND diet | FFQ in 2013; 0–15 MIND score | Approximately 7 years | Global cognitive function and inflammatory biomarker mediation | Higher MIND adherence was associated with lower IL-1RA, IL-6, IL-10, sTNFR-1, CRP, cystatin C, neutrophils, and WBC and higher albumin. Mediation proportions were 11.6% for cystatin C, 10.3% for sTNFR-1, 5.0% for IL-6, 3.7% for WBC, and 3.6% for IL-10; combined mediation was 13.3% (P=.024). |
| Féart et al. (2009) | France | Prospective population-based cohort study (Three-City, Bordeaux) | Community-dwelling adults aged ≥65 years | 1,410 | Mean 75.9 years (range 67.7–94.9) | Mediterranean diet (MeDi) | Food-frequency questionnaire plus 24-hour dietary recall; 0–9 MeDi score | Median 4.1 years; examinations over 5 years | Change in MMSE, IST, BVRT, and FCSRT; incident dementia | Per 1-point higher MeDi score, MMSE error progression was lower (β=−0.006, 95% CI −0.010 to −0.0003; P=.04). Highest versus lowest adherence among dementia-free participants was associated with MMSE (β=−0.03, 95% CI −0.05 to −0.001; P=.04) and FCSRT (β=0.21, 95% CI 0.008–0.41; P=.04), but not incident dementia (HR=1.12, 95% CI 0.60–2.10; P=.72). |
| Gardener et al. (2015) | Australia | Prospective cohort study (Australian Imaging, Biomarkers and Lifestyle study) | Cognitively healthy adults aged ≥60 years | 527 | Older adults aged ≥60 years | Australian Mediterranean diet; Western and prudent patterns | Cancer Council of Victoria FFQ; AusMeDi score and data-driven dietary patterns | 36 months | Global cognition and six cognitive-domain composites | Higher AusMeDi adherence was associated with better executive function at 36 months among APOE ε4 carriers (P<.01). Higher Western-pattern adherence was associated with greater visuospatial decline in noncarriers (P<.01); other associations were not significant. |
| Ghosh et al. (2020) | United Kingdom, France, the Netherlands, Italy, and Poland | Secondary analysis of a multicentre randomized controlled dietary intervention (NU-AGE) | Community-dwelling older adults enrolled in NU-AGE | 612 with paired microbiome data (289 control; 323 intervention) | 65–79 years | NU-AGE Mediterranean-style diet | 7-day food records; NU-AGE dietary adherence score | 12 months | Babcock Memory Score and Constructional Praxis; frailty and health-status measures | Diet-enriched microbial taxa were positively associated with Babcock memory and Constructional Praxis and negatively associated with frailty and inflammatory markers; no single cognitive HR/OR/RR was reported. |
| Hayden et al. (2017) | United States | Prospective cohort study (Women’s Health Initiative Memory Study) | Women aged 65–79 years | 7,085 | 65–79 years | Dietary Inflammatory Index (DII) | Baseline WHI/Block FFQ; DII categorized into four groups | Mean 9.7 years | Global cognitive decline and incident MCI or all-cause dementia | Compared with the most anti-inflammatory group, the most pro-inflammatory group had higher risk of MCI/dementia (HR=1.27, 95% CI 1.06–1.52); intermediate groups HR=1.01 (0.86–1.20) and 0.99 (0.82–1.18). Higher DII was also associated with greater cognitive decline. |
| Huang et al. (2023) | China | Prospective cohort study (China Health and Nutrition Survey) plus meta-analysis | Adults aged ≥55 years | 4,066; meta-analysis 26,103 participants | Mean 61.7 years | Modified MIND diet | Three consecutive 24-hour recalls plus household food weighing across repeated survey rounds; 0–12 modified MIND score | Median 3 years; maximum 9 years | Global cognitive function and cognitive decline | Each 3-point higher MIND score was associated with higher global cognition (β=.11, 95% CI .06–.16; P for trend<.001). Meta-analysis per 1-SD higher score: global cognition β=.042 (95% CI .020–.065), but annual decline β=.014 (95% CI −.010 to .037) was not significant. |
| Jennings et al. (2024) | United Kingdom | Three-arm randomized feasibility/effectiveness trial (MedEx-UK) | Cognitively intact older adults at elevated dementia risk | 104 randomized | Mean 67.4±4.6 years (range 57–76) | Mediterranean diet alone or Mediterranean diet plus physical activity | 14-item MEDAS plus five nonconsecutive Intake24 24-hour recalls | 24-week intervention plus 24-week maintenance phase (48 weeks total) | General cognition, processing speed, executive function, memory, and spatial navigation | At 24 weeks, Mediterranean-diet groups improved general cognition by 0.22 (95% CI 0.05–0.35) and memory by 0.31 (95% CI 0.10–0.51) versus control; these cognitive differences were not maintained at 48 weeks. MEDAS improved by 3.7 points (2.9–4.5) at 24 weeks and 2.7 (1.6–3.7) at 48 weeks. |
| Karstens et al. (2019) | United States | Cross-sectional observational study | Cognitively healthy older adults | 82 | Mean approximately 68.8 years | Mediterranean diet | Block 2005 FFQ; Mediterranean-diet score categorized above/below the median | Cross-sectional | Learning and memory; information processing/executive function; hippocampal and dentate-gyrus volumes; WMH | Higher Mediterranean-diet adherence was associated with better learning/memory and larger dentate-gyrus volumes; information processing/executive function and WMH were not significantly associated. |
| Keenan et al. (2020) | United States | Post hoc observational analysis of two randomized supplement-trial cohorts (AREDS and AREDS2) | Adults with age-related macular degeneration enrolled in AREDS/AREDS2 | 7,756 | 50–85 years at trial enrollment | Alternative Mediterranean diet (aMED) | 90-item modified Block FFQ (AREDS) or 131-item Harvard FFQ (AREDS2); aMED score | Cognitive assessments over approximately 5–10 years | Cognitive impairment and cognitive-test scores | Highest versus lowest aMED tertile was associated with lower odds of impairment: AREDS 3MS OR=0.36 (P=.0001) and composite OR=0.56 (P=.001); AREDS2 TICS-M OR=0.56 and composite OR=0.48 (both P<.0001). Overall aMED was not associated with slower decline; higher fish intake was associated with slower decline (P=.019). |
| Luciano et al. (2017) | Scotland, United Kingdom | Prospective cohort study | Lothian Birth Cohort 1936 participants with repeated brain MRI | 401 for total brain volume; 398 for gray matter; 323 for cortical thickness | Approximately 73 years at baseline MRI and 76 years at follow-up | Mediterranean-type diet (MeDi) | 168-item Scottish Collaborative Group food-frequency questionnaire at age 70; 0–9 MeDi score | 3 years | Change in total brain volume, gray-matter volume, and cortical thickness; MMSE available | Lower MeDi adherence was associated with greater 3-year total brain-volume loss, explaining 0.5% of variance (P<0.05); gray matter, cortical thickness, and cross-sectional associations were not significant. |
| Marseglia et al. (2018) | United Kingdom, France, the Netherlands, Italy, and Poland | Multicentre randomized controlled trial (NU-AGE) | Independently living adults aged 65–79 years | 1,279 randomized (638 control; 641 intervention) | 65–79 years | Individually tailored NU-AGE Mediterranean-style diet | 7-day food records and NU-AGE diet-adherence score | 12 months | Global cognition and five cognitive domains | The randomized between-group differences in cognitive change were not significant. In adherence analyses, high versus low adherence was associated with global cognition (β=.20, 95% CI .004–.39; P=.046) and episodic memory (β=.15, 95% CI .02–.28; P=.025). |
| Martínez-Lapiscina et al. (2013) | Spain | Randomized cognitive substudy within the Prevención con Dieta Mediterránea trial (Navarra) | Community-dwelling older adults at high vascular risk | 522 | Mean 74.6±5.7 years at cognitive evaluation | Mediterranean diet supplemented with extra-virgin olive oil; Mediterranean diet supplemented with mixed nuts; low-fat control | 137-item food-frequency questionnaire; 14-item Mediterranean-diet adherence questionnaire; randomized intervention assignment | Mean 6.5 years | Global cognition; mild cognitive impairment and dementia diagnoses | Adjusted end-of-follow-up Mini-Mental State Examination and Clock Drawing Test scores were higher in both Mediterranean-diet groups than in the control group. Baseline cognition was not measured, so within-person cognitive change could not be estimated. |
| McGrattan et al. (2021) | United Kingdom (Northern Ireland) | Mixed-methods pilot randomized controlled trial | Adults with mild cognitive impairment (MCI) or subjective cognitive impairment (SCI) | 20 randomized (15 MCI; 5 SCI); 10 completed | MCI: 76.5±7.0 years; SCI: 67.2±7.9 years | Mediterranean diet (THINK-MED education intervention) | Adapted 14-item Mediterranean Diet Score/MEDAS-type screener | 12 months | Feasibility, Mediterranean-diet adherence, and multidomain cognitive performance | Diet adherence improved in intervention participants, but recruitment, 50% attrition, and missing cognitive data precluded a reliable estimate of cognitive effectiveness. |
| van Lent et al. (2025) | United States | Prospective cohort study (Framingham Heart Study Offspring Cohort) | Dementia-free older adults | 1,487 | Mean 69±6 years | Dietary Inflammatory Index (DII) | Repeated FFQs at up to three examinations; averaged DII based on 36 components | Median 13.1 years; maximum 22.3 years | Incident all-cause dementia and Alzheimer disease (AD) | Each 1-unit higher averaged DII was associated with all-cause dementia (HR=1.21, 95% CI 1.10–1.33; P<.001) and AD (HR=1.20, 95% CI 1.07–1.34; P=.001). |
| Morris et al. (2015) | United States | Prospective cohort study (Memory and Aging Project) | Community-dwelling older adults initially free of dementia | 960 | Mean 81.4 years | MIND diet | Validated semiquantitative FFQ; 0–15 MIND score | Mean 4.7 years | Global cognition and five cognitive domains | Higher MIND score was associated with slower global cognitive decline (β=.0092 per point; P<.0001). The estimated difference between highest and lowest adherence was equivalent to approximately 7.5 years of age-related cognitive difference. |
| Ni et al. (2025) | Spain | Prospective cohort analysis within PREDIMED-Plus | Older adults with overweight/obesity and metabolic syndrome | 746 | Mean 65±5 years | Mediterranean diet | 14-item MEDAS; baseline stool-derived Mediterranean-diet gut microbial signature | 6 years | Global cognition, executive function, and general cognition | Higher MEDAS adherence (P=.007) and a Mediterranean-diet gut microbial signature (P=.036) were independently associated with slower global cognitive decline. The microbial signature was associated with preserved executive function (P=.049), and MEDAS with general cognition (P=.028); Eggerthella abundance was associated with executive decline (FDR<.05). |
| Pelletier et al. (2015) | France | Prospective neuroimaging cohort study (Three-City, Bordeaux) | Older community-dwelling adults without dementia | 146 | Approximately 73 years at dietary assessment | Mediterranean diet | FFQ plus 24-hour recall; 0–9 Mediterranean score | Approximately 9 years to MRI | White-matter microstructure, gray-matter volume, and cognitive performance | Higher Mediterranean-diet adherence was associated with preserved white-matter microstructure, and the implicated tracts were related to better cognition; no association with gray-matter volume was detected. |
| Remie et al. (2026) | The Netherlands | Exploratory cross-sectional study | Older adults aged 60–75 years at increased risk of cognitive decline | 88 | 60–75 years | MIND-NL diet | Validated FFQ; MIND-NL adherence score | Cross-sectional | Global cognitive functioning and brain neuroinflammatory metabolites | MIND-NL adherence was not independently associated with cognition or neuroinflammatory metabolites. Higher adherence attenuated associations of systemic inflammation with neuroinflammation (interaction β=−.22; P=.04) and cognition (β=.22; P=.02), and of barrier permeability with neuroinflammation (β=−.28; P=.05). Within low adherence, neuroinflammation mediated the inflammation–cognition relation (indirect effect .427, 95% CI .072–.891; P=.04). |
| Samieri et al. (2013) | United States | Prospective cohort study (Women’s Health Study) | Women aged ≥65 years | 6,174 | ≥65 years | Alternative Mediterranean diet (aMED) | Semiquantitative FFQ; 0–9 aMED score | Diet approximately 5 years before initial cognition; three cognitive assessments over 4 years | Global cognition and verbal-memory trajectories | aMED adherence was not associated with global-cognition or verbal-memory trajectories (P for interaction=.26 and .40) or average levels (P for trend=.63 and .44). A higher MUFA:SFA ratio was associated with more favorable trajectories (P=.03 and .05). |
| Sawyer et al. (2024) | United States | Prospective biracial cohort study (REGARDS) | Black and White adults without baseline cognitive impairment | 14,145 | Mean 64±9 years | MIND diet | Block 98 FFQ (>150 items); 15-component MIND score | Approximately 10 years; up to 9 cognitive assessment occasions | Incident cognitive impairment and longitudinal cognitive trajectory | Each higher MIND-score point was associated with lower odds of incident impairment (OR=0.96, 95% CI 0.93–0.99; P=.02). The association was present in women (OR=0.92, 0.89–0.96; P<.001) but not men (OR=1.01, 0.97–1.06; P=.64). Higher adherence was associated with slower decline in Black (β=0.04, SE=.007; P<.001) and White participants (β=0.03, SE=.004; P<.001). |
| Scarmeas et al. (2006) | United States | Prospective community-based cohort study (Washington Heights–Inwood Columbia Aging Project) | Community-dwelling adults without dementia | 2,258 | Mean 77.2±6.6 years in the analytic cohort | Mediterranean diet (MeDi) | Willett semiquantitative FFQ; 0–9 MeDi score | Mean 4.0±3.0 years (range 0.2–13.9) | Incident Alzheimer disease | Each 1-point higher MeDi score was associated with lower AD risk (HR=.91, 95% CI .83–.98; P=.015). Compared with the lowest tertile, middle adherence HR=.85 (95% CI .63–1.16) and highest adherence HR=.60 (95% CI .42–.87); P for trend=.007. |
| Shannon et al. (2023) | United Kingdom | Prospective cohort study (UK Biobank) | Adults aged ≥60 years without dementia | 60,298 | Mean 63.8±2.7 years | Mediterranean diet (MEDAS continuous and Mediterranean Pyramid scores) | Up to five Oxford WebQ self-administered 24-hour dietary recalls | Mean 9.1 years | Incident all-cause dementia | Highest versus lowest adherence was associated with lower dementia risk for the continuous MEDAS score (HR=.77, 95% CI .65–.91); the Pyramid score estimate was HR=.86 (95% CI .73–1.02). No consistent interaction with polygenic risk was observed. |
| Shi et al. (2023) | United Kingdom | Prospective cohort study (UK Biobank) | Adults without dementia at baseline | 166,377 | Mean 55.91±7.98 years (baseline range 37–73) | Dietary Inflammatory Index (DII) | Oxford WebQ 24-hour recall; DII derived from 29 available food/nutrient parameters | Median 9.46 years | Incident all-cause dementia and subtypes; WMH and hippocampal gray-matter volume | All-cause dementia incidence increased 4.6% per DII unit (HR=1.046). Highest versus lowest DII quartile HR=1.249 (95% CI 1.052–1.483; P=.011). Above DII 1.30, AD risk increased (HR=1.391, 95% CI 1.085–1.784; P=.009; P nonlinear=.003). Each DII unit corresponded to 73.30 mm³ higher WMH (P=.006) and lower hippocampal gray matter (β=−7.617, 95% CI −13.986 to −1.248; P=.019). |
| Tangney et al. (2011) | United States | Prospective population-based cohort study (Chicago Health and Aging Project) | Black and White community-dwelling adults aged ≥65 years | 3,790 | Mean 75.4±6.2 years | Mediterranean diet (MedDiet); Healthy Eating Index 2005 comparator | 139-item FFQ; 0–55 MedDiet score | Mean 7.6 years | Rate of change in global cognition | Higher MedDiet adherence was associated with slower decline (β=0.0014 per score point, SEE=0.0004; P=.0004); HEI-2005 was not associated with decline. |
| Titova et al. (2013) | Sweden | Observational cohort with a 5-year exposure–outcome lag | Cognitively healthy older adults in the Prospective Investigation of the Vasculature in Uppsala Seniors | 194 | Diet at age 70; cognition/MRI at age 75 | Mediterranean diet | 7-day food diary; Mediterranean-diet adherence score | 5 years | Global cognitive screening and brain volume | The overall Mediterranean score was not clearly associated with cognition or brain volume. Lower meat intake was associated with better 7-Minute Screen performance (P=.001) and greater total brain volume (P=.03). |
| Valls-Pedret et al. (2015) | Spain | Randomized cognitive substudy within the Prevención con Dieta Mediterránea trial (Barcelona-North) | Cognitively healthy older adults at high cardiovascular risk | 447 at baseline; 334 completed follow-up | Mean 66.9 years at baseline | Mediterranean diet supplemented with extra-virgin olive oil; Mediterranean diet supplemented with mixed nuts; low-fat control | 137-item food-frequency questionnaire; 14-item Mediterranean Diet Adherence Screener; intervention assignment and adherence biomarkers | Median 4.1 years among completers (range 1.0–8.8) | Memory, frontal-function, and global-cognition composites; incident mild cognitive impairment | Adjusted cognitive change favored the olive-oil Mediterranean-diet arm for frontal and global composites and the nut-supplemented arm for the memory composite versus control. Attrition was substantial and differed across groups. |
| Vercambre et al. (2012) | United States | Prospective cohort study nested in the Women’s Antioxidant Cardiovascular Study | Women aged ≥65 years with cardiovascular disease or ≥3 coronary risk factors | 2,504 | ≥65 years | Mediterranean-style diet | 116-item Willett semiquantitative FFQ; 0–9 Mediterranean score | Four cognitive assessments over 5.4 years; diet assessed 3.5 years before initial cognition | Global cognition, verbal memory, and category fluency | Mediterranean-style diet adherence was not associated with subsequent 5-year cognitive change, and no prespecified effect modification was detected. |
| Wagner et al. (2023) | United States | Longitudinal clinical–pathologic cohort study (Memory and Aging Project) | Deceased older adults with annual cognition and postmortem neuropathology | 578 | Mean 84.1±5.8 years at first dietary assessment | MIND diet | Validated semiquantitative FFQ; 0–15 MIND score | Annual follow-up for up to 23 years before death | Cognitive resilience to Alzheimer and other neuropathologies | Highest versus lowest MIND tertile was associated with higher CRLevel (mean difference=.34, 95% CI .14–.55) and CRSlope (.27, 95% CI .05–.48) after adjustment for neuropathologies. |
| Wengreen et al. (2013) | United States | Prospective population-based cohort study (Cache County Study) | Adults aged ≥65 years | 3,831 enrolled; 3,580 in analytic FFQ sample | ≥65 years | DASH and Mediterranean-style diets | 142-item self-administered FFQ; rank-based DASH and Mediterranean scores | Up to 11 years; 3MS assessed up to four times | Average global cognition and change over time | Highest versus lowest quintile participants averaged 0.97 higher 3MS points for DASH (P=.001) and 0.94 for Mediterranean diet (P=.001); differences were maintained over 11 years. Whole grains and nuts/legumes were associated with 1.19 and 1.22 higher points (both P<.001). |
| de Crom et al. (2022) | The Netherlands | Prospective population-based cohort study (Rotterdam Study) | Two largely nonoverlapping population cohorts free of dementia at baseline | 5,375 (RS-I) and 2,861 (RS-II) | Older adults; cohort-specific baseline ages | MIND diet | Validated FFQs; 15-component MIND score | Mean 15.6 years (RS-I) and 5.9 years (RS-II) | Incident all-cause dementia | Per 1-SD higher MIND score, dementia risk was lower during the first 7 years in RS-I (HR=.85, 95% CI .74–.98) and RS-II (HR=.76, 95% CI .66–.87), but the association attenuated with longer follow-up. |

*Note.* AD, Alzheimer disease; aMED, alternative Mediterranean diet; CERAD-NB, Consortium to Establish a Registry for Alzheimer’s Disease Neuropsychological Battery; CMD, cardiometabolic disease; DII, Dietary Inflammatory Index; E-DII, energy-adjusted Dietary Inflammatory Index; EDII, Empirical Dietary Inflammatory Index. Duggan et al. used EDII to denote the energy-adjusted Dietary Inflammatory Index; to avoid confusion with the Empirical Dietary Inflammatory Index, this review standardizes the former as E-DII. FFQ, food-frequency questionnaire; MEDAS, Mediterranean Diet Adherence Screener; MCI, mild cognitive impairment; MeDi, Mediterranean diet score; MIND, Mediterranean–DASH Intervention for Neurodegenerative Delay; MMSE, Mini-Mental State Examination; MRI, magnetic resonance imaging; 3MS, Modified Mini-Mental State Examination; TICS-M, Telephone Interview for Cognitive Status–Modified; WMH, white-matter hyperintensity. Statistical values were not imputed when absent from the source. All abbreviations used in the supplementary materials are listed in the Supplementary Abbreviations section.

Supplementary Table S3. Dietary patterns and inflammatory characteristics

| **Dietary pattern** | **Components** | **Dietary characteristics** | **Anti-inflammatory mechanisms** | **Representative studies** |
| --- | --- | --- | --- | --- |
| Mediterranean diet | Olive oil; vegetables; fruits; legumes; nuts; fish; whole grains; moderate dairy/alcohol in some scores; limited red/processed meat and sweets | Predominantly plant-forward, high in monounsaturated fats, fiber, polyphenols, carotenoids, and marine n-3 fatty acids; low in refined and processed foods | May reduce systemic cytokine signaling and oxidative burden, improve endothelial/neurovascular function, support gut-microbial diversity and short-chain-fatty-acid production, and reduce amyloid/tau-related neurodegenerative processes. | Scarmeas et al. (2006); Féart et al. (2009); Tangney et al. (2011); Martínez-Lapiscina et al. (2013); Valls-Pedret et al. (2015); Marseglia et al. (2018); Ballarini et al. (2021); Dove et al. (2024); Ni et al. (2025) |
| MIND diet | Green leafy vegetables; other vegetables; berries; nuts; beans; whole grains; fish; poultry; olive oil; wine (where applicable); limited red meat, butter/margarine, cheese, pastries/sweets, and fried/fast food | Hybrid of Mediterranean and DASH patterns with explicit emphasis on foods linked to brain health and flexible adherence scoring | May lower systemic inflammatory signaling, improve vascular/metabolic risk, increase antioxidant and flavonoid exposure, and enhance cognitive resilience despite accumulated neuropathology. | Morris et al. (2015); de Crom et al. (2022); Chen et al. (2023); Wagner et al. (2023); Barnes et al. (2023); Sawyer et al. (2024); Agarwal et al. (2024); Fei et al. (2025) |
| DASH diet | Fruits; vegetables; whole grains; low-fat dairy; nuts and legumes; fish/poultry; low sodium; limited red/processed meat, sweets, and sugar-sweetened beverages | Nutrient-dense, low-sodium pattern designed for blood-pressure reduction and vascular-risk control | Potential cognitive benefit is primarily mediated through blood-pressure control, reduced endothelial dysfunction, improved cerebral perfusion, and lower cardiometabolic inflammatory burden. | Wengreen et al. (2013) |
| Dietary Inflammatory Index (DII) | Literature-derived scoring framework combining pro- and anti-inflammatory dietary parameters; positive scores indicate more pro-inflammatory intake | Quantifies inflammatory potential rather than adherence to a prespecified food pattern; component availability varies by dietary instrument | Lower scores reflect dietary exposures expected to reduce CRP/cytokine signaling, vascular injury, white-matter damage, and inflammation-associated neurodegeneration. | Hayden et al. (2017); Charisis et al. (2021); Shi et al. (2023); Dove et al. (2024); van Lent et al. (2025) |
| Energy-adjusted Dietary Inflammatory Index (E-DII) | DII parameters standardized per unit of energy intake (commonly per 1,000 kcal) | Reduces confounding by total energy intake while retaining the literature-derived inflammatory weighting | Same conceptual pathways as DII; energy standardization improves comparability of dietary inflammatory density. | Duggan et al. (2023; reported as EDII in the original study) |
| Empirical Dietary Inflammatory Index/Pattern (EDII/EDIP) | Food-group pattern empirically weighted against circulating inflammatory biomarkers; nomenclature differs across cohorts | Food-based, data-derived inflammatory score that may better represent culturally specific combinations of foods | Captures dietary combinations associated with IL-6, CRP, TNF-receptor signaling, oral/gut dysbiosis, and downstream cognitive effects. | Chou et al. (2025) (termed EDII in the source) |

*Note.* Mechanistic descriptions summarize biologically plausible pathways supported by the measured findings in the included studies; they do not imply that every pathway was directly tested in every study. DII, Dietary Inflammatory Index; E-DII, energy-adjusted Dietary Inflammatory Index; EDII, Empirical Dietary Inflammatory Index; EDIP, Empirical Dietary Inflammatory Pattern; DASH, Dietary Approaches to Stop Hypertension; MIND, Mediterranean–DASH Intervention for Neurodegenerative Delay. Duggan et al. used EDII to denote the energy-adjusted Dietary Inflammatory Index; this review standardizes that exposure as E-DII.

Supplementary Table S4. Cognitive outcomes and assessment instruments

| **Study** | **Cognitive outcome** | **Assessment tool** | **Definition** | **Time point** |
| --- | --- | --- | --- | --- |
| Agarwal et al. (2024) | Change in global cognition | Composite of East Boston immediate and delayed recall, MMSE, and Symbol Digit Modalities Test | Standardized global cognitive score across repeated assessments | At least two assessments during follow-up |
| Ballarini et al. (2021) | Memory, mediotemporal gray-matter volume, CSF Aβ42/40, and phosphorylated tau | Neuropsychological memory composite; structural MRI; CSF Aβ42/40 and p-tau181 assays | Cross-sectional diet associations with memory and Alzheimer-disease biomarkers | Baseline |
| Barnes et al. (2023) | Global cognition, four cognitive domains, and MRI brain outcomes | 12-test neuropsychological battery; structural brain MRI | Annual change in standardized global cognition and domain scores; MRI volume changes | Baseline and annual cognition through year 3; MRI baseline and year 3 |
| Charisis et al. (2021) | Incident dementia | Comprehensive neuropsychological evaluation and expert clinical consensus | Incident all-cause dementia | Baseline and follow-up evaluation |
| Chen et al. (2023) | Incident all-cause dementia | Cohort-specific clinical/record-based dementia ascertainment | Incident dementia during cohort follow-up | Prospective cohort surveillance |
| Chou et al. (2025) | Global cognition, memory, attention, executive function, and verbal fluency | MoCA–Taiwanese version; WMS-III Logical Memory and Digit Span; Trail Making Tests A/B; category verbal fluency | Continuous domain performance and cognitive impairment defined as MoCA-T<24 | Baseline and biennially |
| de Crom et al. (2022) | Incident all-cause dementia | Continuous electronic screening and clinical dementia work-up | Incident dementia during follow-up | From dietary baseline through 2018 |
| Dove et al. (2024) | Incident dementia; gray-matter volume and white-matter hyperintensity (WMH) volume | Linked medical records for dementia; structural brain MRI | Incident all-cause dementia and standardized MRI markers of brain aging | Dietary assessments at baseline/repeats; dementia follow-up through January 20, 2022; MRI during follow-up |
| Duggan et al. (2023) | Incident MCI or probable dementia and inflammatory/immune proteomic signatures | Annual neuropsychological assessment with central adjudication; Olink plasma proteomics; external biomarker and MRI datasets | Incident cognitive impairment (MCI or probable dementia) and protein associations with E-DII | Baseline diet/plasma and prospective follow-up to 2018 |
| Fei et al. (2025) | Global cognitive function and inflammatory biomarker mediation | 27-point modified Telephone Interview for Cognitive Status; panel of 18 circulating inflammatory/immune biomarkers | Repeated global cognition and proportion of diet–cognition association mediated by biomarkers | Diet 2013; biomarkers 2016; cognition 2016, 2018, and 2020 |
| Féart et al. (2009) | Change in MMSE, IST, BVRT, and FCSRT; incident dementia | MMSE; Isaacs Set Test (IST); Benton Visual Retention Test (BVRT); Free and Cued Selective Reminding Test (FCSRT); expert dementia adjudication | Longitudinal neuropsychological change and incident all-cause dementia | Baseline and 2- and 5-year follow-up waves |
| Gardener et al. (2015) | Global cognition and six cognitive-domain composites | Comprehensive neuropsychological battery | Change in standardized global and domain scores | Baseline, 18 months, and 36 months |
| Ghosh et al. (2020) | Babcock Memory Score and Constructional Praxis; frailty and health-status measures | Babcock Story Recall/Memory test; Constructional Praxis; multidomain clinical assessments | Change in cognitive test performance and associations between diet-responsive microbial taxa, cognition, and frailty | Baseline and 12 months |
| Hayden et al. (2017) | Global cognitive decline and incident MCI or all-cause dementia | Annual 3MS; later TICS-M, Oral Trail Making, and Dementia Questionnaire; central adjudication | Longitudinal cognitive score and incident centrally adjudicated cognitive impairment | Annual assessments |
| Huang et al. (2023) | Global cognitive function and cognitive decline | Immediate and delayed 10-word recall, backward counting, and serial-7 subtraction; standardized global score | Global cognitive z score and annual change | Repeated CHNS cognitive waves |
| Jennings et al. (2024) | General cognition, processing speed, executive function, memory, and spatial navigation | Extended neuropsychological test battery including Digit Symbol Substitution, Trail Making Tests, verbal fluency, Digit Span, paired-associate tests, Rey Auditory Verbal Learning Test, Hayling test, and spatial-navigation tasks | Standardized domain and general-cognition composite scores | Baseline, 24 weeks, and 48 weeks |
| Karstens et al. (2019) | Learning and memory; information processing/executive function; hippocampal and dentate-gyrus volumes; WMH | Neuropsychological composite scores and structural brain MRI | Cross-sectional cognitive-domain and neuroimaging phenotypes | Single assessment |
| Keenan et al. (2020) | Cognitive impairment and cognitive-test scores | Modified Mini-Mental State Examination (3MS), Telephone Interview for Cognitive Status–Modified (TICS-M), and composite cognitive batteries | 3MS<80, TICS-M<30, and study-specific composite impairment thresholds; longitudinal score change | AREDS late follow-up; AREDS2 at baseline and approximately 2, 4, and 10 years |
| Luciano et al. (2017) | Change in total brain volume, gray-matter volume, and cortical thickness; MMSE available | Structural brain MRI; Mini-Mental State Examination (MMSE) | Longitudinal percentage change in MRI-derived brain measures | MRI at ages 73 and 76; diet at age 70 |
| Marseglia et al. (2018) | Global cognition and five cognitive domains | Consortium to Establish a Registry for Alzheimer’s Disease Neuropsychological Battery (CERAD-NB) and MMSE | Change in global cognition, episodic memory, perceptual speed, executive function, verbal ability, and constructional praxis | Baseline and 1 year |
| Martínez-Lapiscina et al. (2013) | Global cognition; secondary mild cognitive impairment and dementia diagnoses | Mini-Mental State Examination; Clock Drawing Test; clinical-record review with adjudication and neurologist reassessment where indicated | End-of-follow-up Mini-Mental State Examination (0–30) and Clock Drawing Test (0–7) scores; incident clinical diagnoses | Single cognitive assessment after a mean 6.5 years of intervention; no baseline cognitive assessment |
| McGrattan et al. (2021) | Feasibility, Mediterranean-diet adherence, and multidomain cognitive performance | Cambridge Neuropsychological Test Automated Battery (CANTAB: MOT, RTI, PAL, PRM, DMS, SWM, and RVP); MoCA used in screening | Change in computerized cognitive-test performance; recruitment, retention, and intervention feasibility | Baseline and 12 months |
| van Lent et al. (2025) | Incident all-cause dementia and Alzheimer disease (AD) | Continuous dementia surveillance; DSM-IV and NINCDS-ADRDA diagnostic criteria | Expert-adjudicated incident all-cause dementia and AD | Diet averaged before risk follow-up; surveillance through a maximum 22.3 years |
| Morris et al. (2015) | Global cognition and five cognitive domains | Annual 19-test neuropsychological battery | Annual change in standardized global and domain scores | Annual evaluations |
| Ni et al. (2025) | Global cognition, executive function, and general cognition | Repeated neuropsychological composite scores; stool 16S rRNA sequencing | Change in global and domain-specific cognitive composites and associations with microbial signature | Baseline and 2, 4, and 6 years |
| Pelletier et al. (2015) | White-matter microstructure, gray-matter volume, and cognitive performance | Diffusion tensor imaging (DTI), structural MRI, and neuropsychological composites | Voxelwise white-matter integrity, gray-matter volume, and cognitive-domain performance | Diet at baseline; MRI/cognition approximately 9 years later |
| Remie et al. (2026) | Global cognitive functioning and brain neuroinflammatory metabolites | Neuropsychological battery; proton magnetic resonance spectroscopy (¹H-MRS) in dorsolateral prefrontal cortex | Composite cognition; MRS myo-inositol/choline/creatine indices; interaction with systemic inflammation and barrier permeability | Single assessment |
| Samieri et al. (2013) | Global cognition and verbal-memory trajectories | Validated telephone cognitive battery and composite scores | Longitudinal change and average level in global cognition and verbal memory | Initial cognitive assessment followed by two assessments approximately 2 years apart |
| Sawyer et al. (2024) | Incident cognitive impairment and longitudinal cognitive trajectory | Six-Item Screener (SIS), Word List Learning, Word Delayed Recall, Letter Fluency, and Animal Fluency; robust-norms composite | Incident impairment based on robust normative performance and change in composite cognition | Baseline and repeated telephone assessments; second in-home examination approximately 10 years later |
| Scarmeas et al. (2006) | Incident Alzheimer disease | Repeated clinical and neuropsychological evaluation; DSM-III-R dementia and NINCDS-ADRDA AD criteria | Incident probable or possible AD | Approximately every 1.5 years |
| Shannon et al. (2023) | Incident all-cause dementia | Linked hospital and mortality records using ICD coding | Incident all-cause dementia | Prospective follow-up from dietary assessment |
| Shi et al. (2023) | Incident all-cause dementia and subtypes; WMH and hippocampal gray-matter volume | ICD-9/ICD-10 linked diagnoses; structural brain MRI | Incident all-cause, Alzheimer, vascular, and frontotemporal dementia; MRI tissue volumes | Prospective follow-up; MRI imaging subset during follow-up |
| Tangney et al. (2011) | Rate of change in global cognition | Composite of East Boston immediate and delayed recall, MMSE, and Symbol Digit Modalities Test | Standardized global cognitive score assessed approximately every 3 years | Baseline and repeated assessments over a mean of 7.6 years |
| Titova et al. (2013) | Global cognitive screening and brain volume | 7-Minute Screen and structural MRI | Cognitive-screen performance and total/gray/white-matter volumes at age 75 | Diet at 70; outcomes at 75 |
| Valls-Pedret et al. (2015) | Memory, frontal-function, and global-cognition composites; incident mild cognitive impairment | Mini-Mental State Examination; Rey Auditory Verbal Learning Test; Wechsler Memory Scale paired associates; animal fluency; Digit Span; Color Trail Test | Standardized baseline-to-follow-up change scores combined into prespecified memory, frontal-function, and global-cognition composites | Baseline and final assessment; median 4.1 years among completers (range 1.0–8.8) |
| Vercambre et al. (2012) | Global cognition, verbal memory, and category fluency | Five-test validated telephone cognitive battery | Annual rate of change in global and verbal-memory composite scores | Initial cognition 1998–2000 plus three assessments at approximately 2-year intervals |
| Wagner et al. (2023) | Cognitive resilience to Alzheimer and other neuropathologies | Annual 19-test global cognitive composite and standardized postmortem neuropathologic examination | CRLevel (cognitive level unexplained by pathology) and CRSlope (cognitive slope unexplained by pathology) | Annual cognition through death; autopsy at death |
| Wengreen et al. (2013) | Average global cognition and change over time | Modified Mini-Mental State Examination (3MS) | Repeated 100-point 3MS score | Baseline and up to three follow-up assessments over 11 years |

*Note.* Cognitive outcomes include clinical cognition, incident MCI/dementia, cognitive resilience, and structural or molecular brain-aging outcomes when these were the prespecified outcomes of the included article. All abbreviations used in the supplementary materials are listed in the Supplementary Abbreviations section.

Supplementary Table S5. Neuroinflammatory and biological mechanisms

| **Study** | **Biological marker/pathway** | **Measurement** | **Association with diet** | **Cognitive relevance** |
| --- | --- | --- | --- | --- |
| Ghosh et al. (2020) | Gut–brain axis and systemic inflammation | Shotgun metagenomics; CRP and IL-17; inferred microbial metabolite production | NU-AGE diet-responsive taxa were associated with lower CRP and IL-17 and with inferred increases in short-/branched-chain-fatty-acid production and lower secondary bile acids, p-cresols, ethanol, and CO₂. | Diet-enriched taxa were positively associated with Babcock memory and Constructional Praxis, supporting a microbiome–inflammation–cognition pathway. |
| Luciano et al. (2017) | Structural brain aging | Longitudinal structural MRI: total brain volume, gray matter, and cortical thickness | Lower Mediterranean-diet adherence predicted greater 3-year total brain-volume loss (0.5% of variance; P<0.05); other MRI measures were not significant. | Total brain-volume preservation is a structural correlate of healthier brain aging, although clinical cognitive effects were not the primary endpoint. |
| Karstens et al. (2019) | Hippocampal neurodegeneration | Structural MRI of hippocampal/dentate-gyrus volumes and WMH | Higher Mediterranean adherence was associated with larger dentate-gyrus volumes; WMH was not significantly associated. | Dentate-gyrus volume was concordant with better learning and memory. |
| Pelletier et al. (2015) | Neurovascular/white-matter pathway | Diffusion tensor imaging and structural MRI | Higher Mediterranean adherence was associated with preserved white-matter microstructure; no association with gray-matter volume was detected. | The preserved white-matter tracts were related to better cognitive performance. |
| Ballarini et al. (2021) | Amyloid/tau pathway and mediotemporal atrophy | CSF Aβ42/40 and p-tau181; structural MRI | Higher Mediterranean adherence was associated with higher Aβ42/40 (β=.003±.001; P=.008), lower p-tau181 (β=−1.96±.68; P=.004), and larger mediotemporal gray-matter volume (P<.05 FWE). | Biomarkers statistically accounted for approximately 40% of the diet–memory association. |
| Dove et al. (2024) | Neurovascular injury and brain atrophy | Structural MRI gray-matter and WMH volumes | Among adults with cardiometabolic disease, anti-inflammatory diet was associated with larger gray-matter volume and smaller WMH burden than pro-inflammatory diet. | The MRI pattern paralleled lower incident dementia risk (HR=.69, 95% CI .55–.88). |
| Shi et al. (2023) | Neurovascular injury and hippocampal atrophy | MRI total WMH and hippocampal gray-matter volume | Each DII unit was associated with 73.30 mm³ higher WMH (P=.006) and lower hippocampal gray matter (β=−7.617, 95% CI −13.986 to −1.248; P=.019). | Higher DII also predicted all-cause and Alzheimer dementia, providing convergent structural and clinical evidence. |
| Remie et al. (2026) | Systemic inflammation, gut-barrier permeability, and neuroinflammatory metabolites | CRP, WBC, neutrophil-to-lymphocyte ratio; LBP, zonulin, and LPS; ¹H-MRS myo-inositol/choline/creatine | MIND-NL was not independently associated with markers, but modified inflammation–neuroinflammation (β=−.22; P=.04) and barrier-permeability–neuroinflammation relations (β=−.28; P=.05). | MIND-NL modified the inflammation–cognition association (β=.22; P=.02); neuroinflammation mediated this relation under low adherence (indirect effect .427, 95% CI .072–.891; P=.04). |
| Duggan et al. (2023) | Systemic inflammatory proteome and neurodegeneration | Olink plasma proteomics; external Aβ42/40, neurofilament light, and MRI atrophy datasets | Fifty-five proteins were associated with E-DII; seven candidates (CXCL10, CCL3, HGF, OPG, CDCP1, NFATC3, and ITGA11) predicted future cognitive impairment, with partial external replication. | Several proteins also correlated with amyloid/neurodegeneration biomarkers or brain atrophy, identifying candidate molecular mediators. |
| Fei et al. (2025) | Systemic inflammation | 18 circulating markers including CRP, IL-6, IL-10, IL-1RA, sTNFR-1, cystatin C, albumin, neutrophils, and WBC | Higher MIND adherence was associated with lower CRP, IL-6, IL-10, IL-1RA, sTNFR-1, cystatin C, neutrophils, and WBC and higher albumin. | Cystatin C, sTNFR-1, IL-6, WBC, and IL-10 jointly mediated 13.3% of the MIND–cognition association (P=.024). |
| Ni et al. (2025) | Gut–brain axis | Stool 16S rRNA sequencing and Mediterranean-diet gut microbial signature | The Mediterranean-diet microbial signature independently predicted slower global decline (P=.036) and preserved executive function (P=.049); Eggerthella predicted executive decline (FDR<.05). | Microbiome associations persisted alongside MEDAS adherence, supporting an independent microbial contribution. |
| Wagner et al. (2023) | Cognitive resilience to neuropathology | Postmortem Alzheimer, vascular, Lewy-body, TDP-43, hippocampal-sclerosis, and other neuropathologic measures | Higher MIND adherence was associated with higher cognitive level and slower decline after accounting for measured neuropathologies (CRLevel difference=.34, 95% CI .14–.55; CRSlope=.27, .05–.48). | The findings support resilience rather than a demonstrated reduction in measured pathology. |
| Barnes et al. (2023) | Structural brain aging (randomized evidence) | Structural MRI at baseline and 3 years | MRI changes did not differ materially between the MIND and active-control groups. | The null MRI result was concordant with the null between-group global-cognition difference (P=.23). |
| Titova et al. (2013) | Structural brain aging | Structural MRI of total, gray-, and white-matter volumes | The overall Mediterranean-diet score was not significantly associated with brain volume; lower meat intake was associated with greater total brain volume | The findings did not demonstrate a consistent association between overall Mediterranean-diet adherence and structural brain aging |

*Note.* Only biomarkers, imaging measures, microbiome features, or neuropathologic outcomes actually measured in the included articles are presented. No included study directly quantified a prespecified oxidative-stress biomarker or microglial activation as the principal mediator; these remain mechanistic evidence gaps. Aβ, amyloid-β; CRP, C-reactive protein; FDR, false-discovery rate; IL, interleukin; LBP, lipopolysaccharide-binding protein; LPS, lipopolysaccharide; MRS, magnetic resonance spectroscopy; NfL, neurofilament light; p-tau, phosphorylated tau; sTNFR-1, soluble tumor necrosis factor receptor 1; WBC, white blood cell count.

Supplementary Table S6. Methodological characteristics and limitations

| **Study** | **Main strength** | **Main limitation** | **Dietary measurement limitation** | **Cognitive assessment limitation** | **Bias concern** |
| --- | --- | --- | --- | --- | --- |
| Agarwal et al. (2024) | Large biracial sample with repeated objective cognitive testing. | Observational study with race-specific attenuation after additional adjustment. | FFQ self-report and baseline dietary scoring. | A single global composite may not identify domain-specific effects. | Residual confounding, socioeconomic confounding, and differential selection/attrition. |
| Ballarini et al. (2021) | Convergent cognitive, MRI, and CSF biomarker measures. | Cross-sectional design and smaller CSF subset. | Single FFQ and cross-sectional dietary score. | Cross-sectional biomarker–cognition associations cannot determine directionality. | Reverse causality, selection into lumbar puncture, and residual confounding. |
| Barnes et al. (2023) | Large, long-duration randomized trial with high retention and MRI substudy. | Mild caloric restriction and dietary counseling in both groups reduced exposure contrast. | Intervention adherence converged partly with improvements in the active control diet; self-reported dietary measures. | Both groups improved, potentially reflecting practice effects and an active comparator. | Contamination/exposure convergence and limited generalizability to cognitively impaired adults. |
| Charisis et al. (2021) | Population-based study with detailed dietary data and expert dementia adjudication. | Short follow-up and limited event count. | Baseline FFQ and literature-derived DII score. | Only 62 incident cases, yielding relatively wide confidence intervals. | Residual confounding, reverse causality, and imprecision. |
| Chen et al. (2023) | Replication across three cohorts and quantitative synthesis. | Between-cohort heterogeneity and observational evidence. | Dietary instruments and score operationalization differed among cohorts. | Dementia ascertainment methods differed across cohorts. | Residual confounding, harmonization error, and selective publication in the meta-analysis. |
| Chou et al. (2025) | Longitudinal domain-specific cognition with oral-health and H. pylori effect-modification analyses. | Single-center Taiwanese cohort and observational design. | Relatively brief FFQ, single baseline exposure, and EDII not validated for vegetarians in this study. | Practice effects and attrition across biennial cognitive assessments. | Residual confounding, selection/loss to follow-up, and multiple interaction tests. |
| de Crom et al. (2022) | Replication in two population cohorts with active dementia surveillance. | Time-varying attenuation suggests possible reverse causality or changing diet. | Single baseline FFQ and cohort-specific dietary measurement. | Clinical dementia incidence does not quantify domain-specific preclinical decline. | Reverse causality, residual confounding, and nonproportional exposure associations over time. |
| Dove et al. (2024) | Large prospective cohort with long follow-up and convergent clinical and neuroimaging outcomes. | Observational design and healthy-volunteer selection in UK Biobank. | A limited number of repeated 24-hour recalls may not represent long-term habitual intake. | Dementia ascertainment relied on linked diagnostic records; MRI was available in a selected subset. | Residual confounding, selection bias, and diagnostic under-ascertainment. |
| Duggan et al. (2023) | Prospective high-dimensional proteomics with long follow-up and external validation. | Protein discovery and validation were heterogeneous across platforms/cohorts. | Single baseline FFQ; E-DII and DII depend on the food and nutrient parameters available. | Outcome combines MCI and probable dementia and was restricted to older women. | Multiple testing, overfitting, residual confounding, and limited demographic generalizability. |
| Fei et al. (2025) | Prospective temporal ordering of diet, biomarker panel, and repeated cognition. | Observational mediation relies on strong no-unmeasured-confounding assumptions. | Single FFQ before biomarker measurement. | Telephone global score provides limited domain specificity. | Residual confounding, measurement error, and potential mediator–outcome confounding. |
| Féart et al. (2009) | Population-based prospective design with repeated domain-specific testing and adjudicated dementia. | Observational design and limited power for incident dementia. | Diet was measured once using self-report and a population-relative score. | Multiple cognitive outcomes increased multiplicity; dementia case numbers were limited. | Residual confounding, multiple testing, and possible healthy-participant selection. |
| Gardener et al. (2015) | Repeated multidomain cognition with APOE-stratified analyses. | Moderate sample and subgroup findings without consistent main effects. | Baseline FFQ and cohort-specific dietary-pattern derivation. | Multiple domains and genotype-stratified analyses increase multiplicity. | Multiple testing, residual confounding, and possible chance subgroup findings. |
| Ghosh et al. (2020) | Multicountry intervention with paired metagenomic data and detailed dietary records. | Secondary, per-protocol-type microbiome analysis; associations between taxa and cognition do not establish mediation. | Dietary intake and adherence were based on self-reported food records; the microbiome analysis used a subset of the randomized cohort. | Cognition was a secondary/exploratory outcome and was represented by selected tests rather than incident MCI or dementia. | Attrition/selection into the paired-sample analysis and multiplicity in microbiome analyses. |
| Hayden et al. (2017) | Long follow-up with annual cognition and centrally adjudicated impairment. | Observational analysis of an all-female trial cohort. | Single baseline FFQ; not all original DII parameters were available. | Cognitive instrument changed during follow-up and outcome combined MCI/dementia. | Residual confounding, measurement change over time, and limited generalizability. |
| Huang et al. (2023) | Repeated, interviewer-administered diet data with household weighing and a non-Western population. | Modified exposure definition and heterogeneous studies in the meta-analysis. | Modified MIND score lacked some original components; repeated short recalls may miss episodically consumed foods. | Brief cognitive battery with limited coverage of executive and visuospatial domains. | Residual confounding and cross-cultural measurement heterogeneity. |
| Jennings et al. (2024) | Theory-based randomized intervention with detailed process evaluation and multidomain cognition. | Small feasibility trial, COVID-related protocol adaptations, and exploratory cognitive outcomes. | Self-reported MEDAS and 24-hour recalls; behavior changes during COVID-19 may have affected intake. | Remote follow-up testing during COVID-19 and missing spatial-navigation measures. | Performance bias, measurement-mode changes, and imprecision. |
| Karstens et al. (2019) | Integrated cognitive and region-specific neuroimaging phenotypes in a diverse sample. | Small cross-sectional sample. | Single self-reported FFQ and dichotomization of dietary adherence. | Cross-sectional cognition cannot establish temporal ordering. | Reverse causality, residual confounding, and low precision. |
| Keenan et al. (2020) | Large sample with repeated cognitive data in two well-characterized cohorts. | Post hoc observational analysis within trials not designed to test Mediterranean-diet effects. | Baseline FFQs differed between AREDS and AREDS2 and are subject to recall and measurement error. | Cognitive instruments and assessment schedules differed between cohorts; severe impairment was underrepresented. | Residual confounding, selection of healthier trial participants, and differential measurement across cohorts. |
| Luciano et al. (2017) | Repeated MRI in a narrow-age birth cohort, reducing age-related heterogeneity. | Observational design, modest effect size, and selective availability of repeated MRI. | Single self-reported FFQ obtained approximately 3 years before baseline MRI. | Primary outcomes were structural MRI measures rather than longitudinal clinical cognitive decline or dementia. | Residual confounding and selection bias from healthier participants completing repeat imaging. |
| Marseglia et al. (2018) | Large multicountry randomized intervention with standardized cognitive testing. | Null intention-to-treat cognitive comparison; positive findings were adherence-based secondary analyses. | Food records and adherence analyses are susceptible to reporting error and post-randomization confounding. | One-year follow-up may be too short for meaningful age-related decline. | Post-randomization adherence selection, attrition, and multiplicity. |
| Martínez-Lapiscina et al. (2013) | Long-duration randomized intervention with blinded cognitive outcome assessment. | Site-specific substudy; only 522 of 969 surviving participants underwent cognitive testing. | Dietary adherence relied on a food-frequency questionnaire and short adherence questionnaire, although intervention contrast was documented. | No baseline cognitive assessment and only two brief global tests; within-person change and domain-specific effects could not be estimated. | Selection into cognitive testing, survivor bias, and imprecision for mild cognitive impairment or dementia outcomes. |
| McGrattan et al. (2021) | Randomized pilot with mixed-methods evaluation in people already experiencing cognitive symptoms. | Very small sample, difficult recruitment, high attrition, and insufficient power for cognitive effects. | Short adherence screener and self-reported dietary behavior. | Very small analyzable sample and incomplete CANTAB follow-up data. | Attrition bias, missing outcome data, and imprecision. |
| van Lent et al. (2025) | Repeated dietary assessment and long, active dementia surveillance. | Predominantly White cohort and observational design. | FFQ self-report; nine original DII parameters were unavailable. | Clinical dementia diagnosis does not capture subtler domain-specific cognitive change. | Residual confounding and limited racial/ethnic generalizability. |
| Morris et al. (2015) | Annual comprehensive cognitive testing and validated dietary instrument. | Single-cohort observational study. | Repeated self-reported FFQ and score based on predefined cut points. | Practice effects and selective survival may affect annual trajectories. | Residual confounding, reverse causality, and healthy-survivor selection. |
| Ni et al. (2025) | Longitudinal cognition with microbiome sequencing in a well-characterized high-risk cohort. | Observational analysis within a trial cohort; microbiome associations do not prove causal mediation. | Brief MEDAS screener; stool microbiome measured at baseline only. | Composite definitions and potential practice effects across repeated testing. | Residual confounding, selection, and high-dimensional microbiome multiplicity. |
| Pelletier et al. (2015) | Long exposure–outcome interval with advanced diffusion MRI. | Small selected imaging subset and observational design. | Single baseline dietary self-report. | Small imaging sample and indirect tract-level links to cognition. | Selection bias, residual confounding, and multiple voxelwise comparisons. |
| Remie et al. (2026) | Integrated diet, systemic inflammation, gut-barrier markers, MRS, and cognition. | Small exploratory cross-sectional sample. | FFQ self-report and locally adapted MIND-NL score. | Cross-sectional cognitive composite cannot establish temporal mediation. | Reverse causality, model overfitting, and multiple testing. |
| Samieri et al. (2013) | Large prospective cohort with repeated cognitive composites and temporal separation from diet. | Female health-professional cohort and no overall aMED association. | Single self-reported FFQ and score based on cohort medians. | Telephone testing and a relatively short cognitive trajectory period. | Residual confounding, limited generalizability, and multiple component analyses. |
| Sawyer et al. (2024) | Large biracial cohort with repeated multidomain cognition and subgroup analyses. | Observational design; sex and race interactions require replication. | Single baseline FFQ and a cohort-adapted MIND score. | Telephone screening and composite definitions differ from formal clinical diagnosis. | Residual confounding, differential attrition, and multiple subgroup testing. |
| Scarmeas et al. (2006) | Community-based multiethnic cohort with repeated expert clinical evaluations. | Observational design and potential loss-to-follow-up differences. | Single baseline FFQ and cohort-median scoring. | Incident AD diagnosis may be affected by diagnostic misclassification and competing mortality. | Residual confounding, reverse causality, and attrition/selection. |
| Shannon et al. (2023) | Large sample with genetic-risk analyses and long follow-up. | Observational UK Biobank sample with limited dietary repeats. | A small number of 24-hour recalls may not capture habitual diet; Mediterranean scores were reconstructed from available items. | Record-linkage diagnosis may under-detect dementia and lacks cognitive-domain data. | Healthy-volunteer selection, residual confounding, and diagnostic misclassification. |
| Shi et al. (2023) | Very large sample with dementia subtypes and MRI correlates. | Relatively young baseline cohort and observational design. | Intermittent 24-hour recalls and DII calculated from only 29 parameters. | Registry-based diagnoses may miss undiagnosed dementia; no repeated neuropsychological testing. | Healthy-volunteer selection, residual confounding, and potential reverse causality. |
| Tangney et al. (2011) | Large biracial population cohort with repeated cognitive testing. | Observational evidence cannot exclude residual confounding. | Self-reported FFQ and score construction adapted to a US population. | Global composite may obscure domain-specific effects. | Dietary measurement error and confounding by socioeconomic and health behaviors. |
| Titova et al. (2013) | Detailed prospective food diary and MRI outcome. | Small sample and primarily cross-sectional outcome analysis at age 75. | One 7-day diary may not represent habitual long-term diet. | Single cognitive assessment at outcome and limited sample size. | Residual confounding, multiple dietary-component tests, and limited precision. |
| Valls-Pedret et al. (2015) | Randomized design with repeated comprehensive cognitive testing and objective adherence biomarkers in a subsample. | Single-center selected substudy with 25.3% attrition that differed across intervention groups. | Food-frequency questionnaire and adherence screener were self-reported; adherence biomarkers were available only in a subsample. | Follow-up intervals varied, and some frontal-function tests were added after recruitment began, reducing complete data for those outcomes. | Differential attrition, selected-substudy participation, protocol deviations in a small number of households, and multiplicity. |
| Vercambre et al. (2012) | Prospective repeated cognitive assessments in a high vascular-risk population. | Homogeneous female health-professional sample and null observational association. | Single baseline FFQ and a long lag between diet and first cognitive assessment. | Telephone battery and ceiling effects in highly educated health professionals. | Residual confounding, selection, and limited generalizability. |
| Wagner et al. (2023) | Long annual cognitive trajectories with comprehensive postmortem pathology. | Highly selected autopsy cohort and observational exposure. | Self-reported FFQ late in life; diet may change during preclinical disease. | Analysis is restricted to decedents who underwent autopsy. | Selection/collider bias, reverse causality, and residual confounding. |
| Wengreen et al. (2013) | Population-based sample with long follow-up and repeated global cognition. | Diet was associated with cognitive level but not clearly with differential slope. | Single baseline FFQ; rank-based scores depend on cohort distribution. | 3MS is a global screening tool with ceiling effects and limited domain specificity. | Residual confounding, survivor bias, and dietary measurement error. |

*Note.* Bias concerns are methodological domains for interpretation, not formal risk-of-bias ratings. FFQ self-report, dietary measurement error, residual confounding, reverse causality, selection/attrition, and limited causal inference are highlighted where applicable. All abbreviations used in the supplementary materials are listed in the Supplementary Abbreviations section.

Supplementary Table S7A. Evidence-informed patient-level nursing actions for dietary cognitive-risk reduction

| **Organizational domain** | **Primary target** | **Potential nurse-supported activities** | **Safety and implementation considerations** |
| --- | --- | --- | --- |
| Screen | Diet quality, nutritional risk, and barriers to healthy eating | Use a locally validated dietary or nutritional screening tool, such as MEDAS where appropriate. Document appetite, swallowing or oral-health problems, food access, cultural preferences, and need for dietitian or medical referral. | Screening should support shared decisions and should not be used to diagnose inflammatory exposure. Adapt delivery to language, health literacy, food availability, and care setting. |
| Stratify | Cognitive status, cardiometabolic risk, frailty, and self-management capacity | Review cognitive symptoms, blood pressure, glycemic status, weight, functional status, frailty, medications, and capacity to shop, cook, and follow a dietary plan. | Do not attribute cognitive symptoms to diet alone. New, progressive, or functionally important impairment requires comprehensive clinical assessment. |
| Plan | Individualized anti-inflammatory dietary pattern and achievable goals | Set one or two measurable food-based goals; adapt Mediterranean- or MIND-oriented principles to comorbidities, preferences, budget, and local foods; use teach-back to confirm understanding. | Avoid unnecessarily restrictive plans. Account for energy and protein requirements, renal or metabolic needs, swallowing status, unintended weight loss, and drug–nutrient interactions. |
| Support | Behavior change, adherence, and caregiver engagement | Provide practical education, substitution plans, barrier problem-solving, reinforcement, caregiver involvement, and planned follow-up contacts. | Sustained support may be needed because adherence can attenuate after active contact ends. Cost, access, caregiver burden, and cognitive limitations may determine feasibility. |
| Monitor and coordinate | Adherence, intake, nutritional status, cognition, function, and referral needs | Reassess goals and barriers; monitor intake, weight, unintended nutritional effects, cognition, and function; coordinate dietitian, physician, dentist, pharmacist, caregiver, and community-resource input as indicated. | Dietary intervention is adjunctive risk reduction and does not replace dementia assessment, cardiometabolic prevention, individualized nutrition care, or established treatment. |

*Note.* This author-derived organizational framework is evidence-informed and proposed; it has not been validated as a nurse-led intervention model. MEDAS, Mediterranean Diet Adherence Screener; MIND, Mediterranean–DASH Intervention for Neurodegenerative Delay.

**Supplementary Table S7B. Service-level requirements for safe clinical implementation**

| **Implementation requirement** | **Recommended approach** | **Suggested indicators** | **Key caution** |
| --- | --- | --- | --- |
| Interprofessional pathway and role clarity | Define screening responsibility, referral thresholds, documentation standards, follow-up ownership, and communication routes among nursing, dietetic, medical, dental, pharmacy, and community services. | Screening completion; referral rate; referral completion; time to specialist review; documented follow-up plan. | A screening pathway without referral capacity may identify needs that the service cannot address. |
| Equitable and culturally adaptable delivery | Provide language-appropriate materials, adapt food examples to local dietary practices, assess food insecurity and affordability, and involve caregivers when self-management capacity is limited. | Reach, uptake, adherence, and retention stratified by socioeconomic, language, cultural, and functional characteristics. | Generic Mediterranean or MIND prescriptions may be inaccessible or culturally inappropriate if not adapted. |
| Implementation evaluation and safety surveillance | Audit reach, adoption, fidelity, adherence, retention, patient-reported acceptability, nutritional adequacy, unintended weight loss, and other adverse nutritional effects. | Reach; adoption; adherence-score change; retention; acceptability; dietitian escalation; unintended weight loss; adverse-event reporting. | Current evidence does not establish that prospective evaluation of these proposed activities prevents dementia; service outcomes should be interpreted as feasibility and risk-reduction indicators. |

*Note.* Suggested indicators should be adapted to local resources and should not be interpreted as a validated core outcome set.

**Supplementary Abbreviations**

The following abbreviations are used in the supplementary materials:

| **Abbreviation** | **Definition** | **Abbreviation** | **Definition** |
| --- | --- | --- | --- |
| **3MS** | Modified Mini-Mental State Examination | **16S rRNA** | 16S ribosomal ribonucleic acid |
| **¹H-MRS** | proton magnetic resonance spectroscopy | **Aβ** | amyloid-beta |
| **Aβ42/40** | amyloid-beta 42/40 ratio | **AD** | Alzheimer disease |
| **aMED** | alternative Mediterranean diet score | **APOE** | apolipoprotein E |
| **APOE ε4** | apolipoprotein E ε4 allele | **AREDS** | Age-Related Eye Disease Study |
| **AREDS2** | Age-Related Eye Disease Study 2 | **ARIC** | Atherosclerosis Risk in Communities Study |
| **AusMeDi** | Australian Mediterranean diet score | **BMI** | body mass index |
| **BVRT** | Benton Visual Retention Test | **CANTAB** | Cambridge Neuropsychological Test Automated Battery |
| **CCL3** | C-C motif chemokine ligand 3 | **CDCP1** | CUB domain-containing protein 1 |
| **CERAD-NB** | Consortium to Establish a Registry for Alzheimer’s Disease Neuropsychological Battery | **CHNS** | China Health and Nutrition Survey |
| **CI** | confidence interval | **CMD** | cardiometabolic disease |
| **COVID-19** | coronavirus disease 2019 | **CRLevel** | cognitive level unexplained by measured neuropathology |
| **CRP** | C-reactive protein | **CRSlope** | cognitive slope unexplained by measured neuropathology |
| **CSF** | cerebrospinal fluid | **CXCL10** | C-X-C motif chemokine ligand 10 |
| **DASH** | Dietary Approaches to Stop Hypertension | **DELCODE** | DZNE-Longitudinal Cognitive Impairment and Dementia Study; DZNE, German Center for Neurodegenerative Diseases |
| **DII** | Dietary Inflammatory Index | **DMS** | Delayed Matching to Sample |
| **DSM-III-R** | Diagnostic and Statistical Manual of Mental Disorders, Third Edition, Revised | **DSM-IV** | Diagnostic and Statistical Manual of Mental Disorders, Fourth Edition |
| **DTI** | diffusion tensor imaging | **E-DII** | energy-adjusted Dietary Inflammatory Index |
| **EDII** | Empirical Dietary Inflammatory Index | **EDIP** | Empirical Dietary Inflammatory Pattern |
| **ESTHER** | Epidemiological Study on Potentials for Prevention, Early Detection, and Optimized Treatment of Chronic Diseases in the Elderly Population | **FCSRT** | Free and Cued Selective Reminding Test |
| **FDR** | false-discovery rate | **FFQ** | food-frequency questionnaire |
| **FOS** | Framingham Offspring Study | **FWE** | familywise error |
| **H. pylori** | Helicobacter pylori | **HEI-2005** | Healthy Eating Index 2005 |
| **HELIAD** | Hellenic Longitudinal Investigation of Aging and Diet | **HGF** | hepatocyte growth factor |
| **HR** | hazard ratio | **HRS** | Health and Retirement Study |
| **I²** | I-squared heterogeneity statistic | **ICD** | International Classification of Diseases |
| **ICD-9** | International Classification of Diseases, Ninth Revision | **ICD-10** | International Classification of Diseases, Tenth Revision |
| **IL** | interleukin | **IL-1RA** | interleukin-1 receptor antagonist |
| **IL-6** | interleukin-6 | **IL-10** | interleukin-10 |
| **IL-17** | interleukin-17 | **IST** | Isaacs Set Test |
| **ITGA11** | integrin subunit alpha 11 | **LBP** | lipopolysaccharide-binding protein |
| **LPS** | lipopolysaccharide | **MEDAS** | Mediterranean Diet Adherence Screener |
| **MedDiet** | Mediterranean diet score | **MedEx-UK** | Mediterranean Diet, Exercise and Dementia Risk in UK Adults |
| **MeDi** | Mediterranean diet score | **MCI** | mild cognitive impairment |
| **MIND** | Mediterranean–DASH Intervention for Neurodegenerative Delay | **MIND-NL** | Netherlands-adapted MIND diet score |
| **MMSE** | Mini-Mental State Examination | **MoCA** | Montreal Cognitive Assessment |
| **MoCA-T** | Taiwanese version of the Montreal Cognitive Assessment | **MOT** | Motor Screening Task |
| **MRI** | magnetic resonance imaging | **MRS** | magnetic resonance spectroscopy |
| **MUFA:SFA** | monounsaturated fatty acid-to-saturated fatty acid ratio | **NFATC3** | nuclear factor of activated T cells 3 |
| **NfL** | neurofilament light | **NINCDS-ADRDA** | National Institute of Neurological and Communicative Disorders and Stroke–Alzheimer’s Disease and Related Disorders Association |
| **NU-AGE** | New Dietary Strategies Addressing the Specific Needs of the Elderly Population for Healthy Ageing in Europe | **OPG** | osteoprotegerin |
| **OR** | odds ratio | **PAL** | Paired Associates Learning |
| **PREDIMED-Plus** | Prevención con Dieta Mediterránea-Plus | **PRM** | Pattern Recognition Memory |
| **p-tau181** | phosphorylated tau at threonine 181 | **REGARDS** | Reasons for Geographic and Racial Differences in Stroke |
| **RR** | risk ratio | **RS-I** | Rotterdam Study cohort I |
| **RS-II** | Rotterdam Study cohort II | **RTI** | Reaction Time |
| **RVP** | Rapid Visual Information Processing | **SCI** | subjective cognitive impairment |
| **SD** | standard deviation | **SE** | standard error |
| **SEE** | standard error of the estimate | **SIS** | Six-Item Screener |
| **sTNFR-1** | soluble tumor necrosis factor receptor 1 | **SWM** | Spatial Working Memory |
| **TDP-43** | transactive response DNA-binding protein 43 kDa | **THINK-MED** | Mediterranean diet and lifestyle education intervention |
| **TICS-M** | Telephone Interview for Cognitive Status–Modified | **TNF** | tumor necrosis factor |
| **UK** | United Kingdom | **US** | United States |
| **WBC** | white blood cell count | **WebQ** | Oxford WebQ 24-hour dietary assessment |
| **WHI** | Women’s Health Initiative | **WII** | Whitehall II study |
| **WMH** | white-matter hyperintensity | **WMS-III** | Wechsler Memory Scale–Third Edition |
